# Building for the Future: A Systematic Review of the Effects of Eccentric Resistance Training on Measures of Physical Performance in Youth Athletes

**DOI:** 10.1007/s40279-023-01843-y

**Published:** 2023-04-25

**Authors:** Thomas E. Bright, Matthew J. Handford, Peter Mundy, Jason Lake, Nicola Theis, Jonathan D. Hughes

**Affiliations:** 1 Cardiff School of Sport and Health Sciences, Cardiff Metropolitan University, Cardiff, UK; 2 School of Sport and Exercise, University of Gloucestershire, Gloucester, UK; 3 School of Sport, Health and Wellbeing, Plymouth Marjon University, Derriford Rd, Plymouth, PL6 8BH UK; 4 Hawkin Dynamics, Inc., Westbrook, ME USA; 5 Department of Sport and Exercise Sciences, Chichester University, Chichester, UK; 6 School of Medical and Health Sciences, Edith Cowan University, Joondalup, WA Australia

## Abstract

**Background:**

Eccentric resistance training is recognised as an effective stimulus for enhancing measures of muscular strength and power in adult populations; however, its value in youth athletes is currently not well understood.

**Objective:**

The aim of this systematic review was to critically appraise the effects of eccentric resistance training on measures of physical performance (i.e. muscular strength, jump, sprint and change of direction) in youth athletes 18 years of age and under.

**Methods:**

Original journal articles published between 1950 and June 2022 were retrieved from electronic search engines of PubMed, SPORTDiscus and Google Scholar’s advanced search option. Full journal articles investigating the acute and chronic effects of eccentric resistance training on measures of physical performance in youth athletes (i.e. a person 18 years of age or under who competes in sport) were included. The methodological quality and bias of each study were assessed prior to data extraction using a modified Downs and Black checklist.

**Results:**

The search yielded 749 studies, of which 436 were duplicates. Three-hundred studies were excluded based upon title and abstract review and a further 5 studies were removed following the modified Downs and Black checklist. An additional 14 studies were identified during backward screening. Accordingly, 22 studies were included in our systematic review. The Nordic hamstring exercise and flywheel inertial training were the most frequently used eccentric resistance training methods in youth athletes. Improvements in physical performance following the Nordic hamstring exercise are dependent upon an increase in the breakpoint angle, rather than training volume (sets and repetitions), and are further elevated with the addition of hip extension exercises or high-speed running. A minimum of 3 familiarisation trials is necessary to elicit meaningful adaptations following flywheel inertial training. Furthermore, an emphasis should be placed upon decelerating the rotating flywheel during the final one to two thirds of the eccentric phase, rather than gradually throughout the entire eccentric phase.

**Conclusions:**

The findings of this systematic review support the inclusion of eccentric resistance training in youth athletes to improve measures of muscular strength, jump, sprint and change of direction performance. The current eccentric resistance training methods are predominantly limited to the Nordic hamstring exercise and flywheel inertial training; however, the efficacy of accentuated eccentric loading to improve jump performance warrants attention in future investigations.

## Key Points

**Table:** 

Nordic hamstring exercise and flywheel inertial training are currently the most used eccentric resistance training methods in youth athletes.
The Nordic hamstring exercise has been shown to provide positive adaptations in measures of physical performance (i.e., sprint speed and change of direction); however, these appear to be mediated by an increase in breakpoint angle (the angle at which the individual can no longer resist the increasing gravitational moment and falls to the floor).
A minimum of 3 familiarisation trials is an essential prerequisite to effectively utilise flywheel inertial training. To ensure the greatest carry over to measures of physical performance after flywheel inertial training, youth athletes should be instructed to gently resist during the first third of the eccentric phase before working maximally to decelerate the rotating flywheel.

## Introduction

With the ever-increasing presence of youth physical development programmes in sports clubs and schools, strength and conditioning (S&C) coaches are looking for novel methods to improve physical performance without causing unnecessary stress, fatigue and risk of injury [1–3]. One of these methods is eccentric resistance training (ERT), for which there is a well-recognised body of evidence demonstrating it to be a highly effective stimulus for enhancing neuromuscular qualities, such as strength and power, albeit primarily in adult populations [4–7]. This method of training exploits the greater force-producing capacity of eccentric compared to isometric and concentric muscle actions [5, 8]. The magnitude of eccentric force enhancement is dependent upon the measurement technique, with forces that range from 10% greater in multi-joint movements [9] to 30% and 80% greater in single-joint movements [10] and isolated muscle actions [11], respectively. Given that developing strength and power in youth athletes is strongly advocated owing to its underpinning of other physical performance capabilities (i.e. jumping, sprint speed and change of direction [COD]) [12–14], it is somewhat surprising that very little research on the effects of ERT in this population has been undertaken.

Previously, there were concerns regarding the potential negative consequences of ERT in children and adolescents. One such concern is the association between unaccustomed ERT and muscle damage or delayed pain, frequently referred to as ‘delayed-onset muscle soreness’ [15, 16]. However, given that a significant amount of a child’s playground and athletic activities will include rapid decelerations, COD, and landing and hopping on 1 or both limbs, exposure to eccentric muscle actions is likely to occur frequently from an early age [17]. Furthermore, plyometric training, which incorporates an eccentric loading stimulus [7], is recommended in children prior to peak-height velocity (PHV; the period in which a child experiences the fastest upward growth in their stature) [12, 14, 18, 19]. Current evidence also supports this notion, suggesting that children and adolescents may experience less severe symptoms following ERT, when compared with adults [16, 20, 21]. The key physiological mechanisms underpinning these preferential responses in youth include lower post-exercise blood lactate levels and faster clearance rates [22–26], an improved blood acid–base regulation [27], lower phosphocreatine depletion and faster resynthesis [28, 29], and faster heart rate recovery [23, 26]. Additional factors such as intramuscular synchronisation [30], agonist–antagonist coactivation [31], degree of volitional activation [32] and a reduced capability to recruit or fully employ type-II motor units may explain the lesser fatigue and muscle damage symptoms experienced by children and adolescents in contrast with adults (for detailed reviews on these topics, please see Drury et al. [33] and Woods et al. [32]).

As originally proposed in 1955 by Erling Asmussen [34], increases in strength observed during growth and maturation are more than can be expected from increases in anthropometry alone. As such, there is a general understanding that neural adaptations (i.e. increases in muscle activation and type-II motor unit recruitment), rather than changes in muscle morphology, are the predominant mechanism responsible for strength enhancements following resistance training interventions in children and younger adolescents [35–37]. As greater force can be developed during eccentric compared to concentric or isometric muscle actions [5], previous research has highlighted the preferential recruitment of fast-twitch muscle fibres and higher-threshold (type-II) motor units during ERT [38, 39]. Research should therefore attempt to understand if ERT interventions lead to superior strength adaptations in youth populations, as compared to traditional resistance training (i.e. equivalent absolute eccentric and concentric loads for a given exercise). Given the contribution of increases in muscle size to strength development following PHV, owing to the naturally heightened levels of circulating testosterone and growth hormone [12, 40], it is also important that future investigations endeavour to understand the adaptations that occur after ERT in youth athletes at this stage of maturity.

To achieve overload during ERT, force, time and displacement can be manipulated through the application of a relatively high force or velocity alongside a smaller or larger range of motion [5, 41, 42]. Studies aiming to apply an eccentric overload in youth athletes have most frequently utilised flywheel inertial training (FIT) [43–45] or the Nordic hamstring exercise (NHE) [46, 47]. Despite promising results stemming from both methods, there are key considerations that warrant discussion. For example, the widespread belief that FIT provides an eccentric overload may be limited because the resistance during the eccentric phase is largely dictated by the effort imparted during the concentric phase [7]. To overcome this, authors have recommended that participants are instructed to free fall during the first third of the eccentric phase before applying a maximal effort to decelerate the rotating flywheel; however, research has demonstrated that several familiarisation trials and technical proficiency are necessary before this strategy is successfully adopted [48, 49].

The NHE is an eccentric-only exercise, placing load on the hamstring muscles whilst they are lengthening, and has been advocated across a number of youth sports to mitigate the risk of hamstring strain injury (HSI) [33, 47, 50–52]. It is proposed that if performed correctly (i.e. gradually lean forward at the slowest possible speed, whilst keeping the shoulders, hips and knees in line with one another throughout the full range of motion), the NHE will elicit an increase in muscle fascicle length (FL) through the addition of more sarcomeres in series [53–55]. This is thought to correspond to a longer muscle length at failure and a modification of the hamstring length–tension relationship acting to alleviate the potential risk of HSI [56]. Despite this being the focus of previous research, investigations have noted improvements in sprint speed, COD and jumping following an NHE intervention in youth athletes [57–59]. Even without performance improvements, the NHE will serve to maintain or enhance player availability, which is important given the impact injury may have on missed training exposure in youth age groups and periods around PHV [60, 61]. A review of the effects of ERT on measures of physical performance in youth athletes is therefore necessary to guide future research that will help to advance the application and outcomes of youth physical development programmes.

The aim of this systematic review was to critically appraise the effects of ERT on measures of physical performance (i.e. muscular strength, jump, sprint speed and COD) in youth athletes 18 years of age and under.

## Methods

This review was conducted according to the Preferred Reporting Items for Systematic Reviews and Meta-Analyses (PRISMA) guidelines for systematic reviews [62].

### Literature Search

**Fig. 1:**
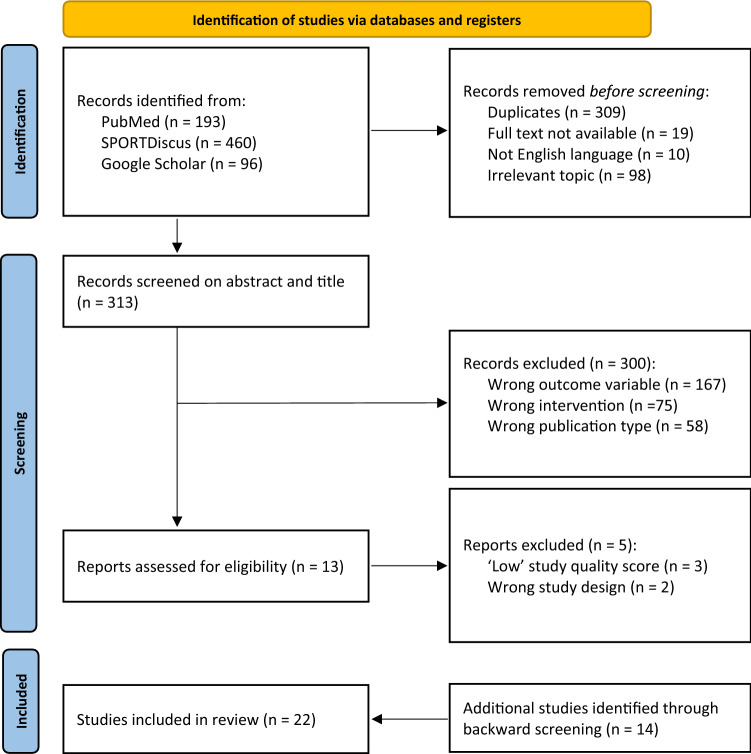
Flow diagram of the selection process

Original journal articles were retrieved from electronic search engines of PubMed, SPORTDiscus and Google Scholar’s advanced search option by 1 reviewer (TB). Figure 1 provides a schematic of the literature search process. Articles published between 1950 and 2022 were considered with the searches beginning and ending on the 4 April, 2022 and the 1 June, 2022, respectively. To supplement the electronic searches, reference lists were cross-checked (TB) for additional research studies that met the inclusion criteria and had not been identified during the initial search process. Combinations of the following search terms were included: ‘youth athlete’, ‘children’, ‘adolescence’, ‘maturation’, ‘eccentric resistance training’, ‘eccentric training’, ‘eccentric exercise’, ‘accentuated eccentric loading’, ‘plyometric training’, ‘stretch–shortening cycle’, ‘jumping’, ‘landing’, ‘Nordic hamstring exercise’ and ‘flywheel inertial training’ in conjunction with the term’s ‘strength’, ‘power’, ‘speed’, ‘change of direction’ and ‘performance’. Boolean operators (AND, OR) were used to concentrate the search terms and avoid excessive quantities of unrelated articles. For example, ‘eccentric resistance training’ AND ‘youth athlete’ OR ‘children’ were combined to capture the relevant training method and sample population only.

### Eligibility Criteria

Full journal articles investigating the acute and chronic effects of ERT in healthy (i.e. the absence of injury of illness during the 4 weeks preceding the study intervention), youth athlete (i.e. 18 years of age and under; competing in sport), human participants were selected for systematic review (Fig. 1). To be considered chronic, studies had to include an intervention that lasted a minimum of 4 weeks. Studies that included testing measures shortly before and/or immediately after a single session were considered acute. Articles were excluded if the criteria were not fulfilled or if an outcome measure of muscular strength, jump, sprint speed or COD performance was not included. A detailed list of the eligibility criteria for this review can be found in Table 1.

**Table 1:** Study eligibility criteria

Number	Criteria
1	Peer-reviewed journal article
2	English language (full text available)
3	Article published between 1950 and June 2022
4	Participants must have been healthy and without injury, disability or illness
5	Participants of 18 years of age and under
6	Analysis of acute and/or chronic effects of eccentric resistance training
7	Intervention clearly outlined
8	Data on at least 1 of the following outcome measures were reported: strength, sprint speed, jump and change of direction performance

### Risk of Bias Assessment

The risk of bias and quality of studies was individually assessed by means of the modified version of the Downs and Black checklist [63], which uses a scoring system to assess the strength of reporting, internal and external validity, and statistical power. Twenty-five items from the original scale were included with a maximum possible score of 24. Assessments were initially carried out by 2 members of the research team (TB and MH) with any disagreements resolved via a third member (JH). Based upon the analyses of a previous systematic review [64], the individual score obtained by each study was divided by 25 and multiplied by 100 to provide a ‘study quality percentage’. The study quality percentage was then classified as high (> 66.7%; low risk of bias), fair (between 50.0% and 66.6%; moderate risk of bias) or low (< 50.0%; high risk of bias) [64, 65]. Studies were excluded if they reported a low study quality percentage score.

## Results and Discussion

A flow diagram of the literature search process is illustrated in Fig. 1. A total of 22 studies were included for systematic review.

### Acute Study Characteristics

Three acute studies were included for review with 51 male participants and a mean age of 16.5 years (range 16.2–17.0 years). The participants in these studies were selected from soccer (2 studies; 66.7%) and rugby union (1 study; 33.3%) teams. Two of the 3 studies included a control group for analysis. The chosen ERT methods included: FIT (2 studies; 66.7%) and accentuated eccentric loading (AEL) [1 study; 33.3%]. The acute study findings are displayed in Table 2.

**Table 2:** Acute eccentric resistance training studies

Study	Objective	Protocol	Participants	Relevant findings	Conclusion
de Hoyo et al. [76]	To analyse the acute effects of ERT on physical performance and kinetic parameters during COD tasks	EXP group = 5 min stationary cycling followed by 4 sets of 6 repetitions of half-squats on a FIT device (knee angle at bottom of half-squat = 90°) with 120-s recovery between sets. Participants were instructed to perform the concentric phase as fast as possible CON group = 5 min stationary cycling	*n* = 20 male soccer participants, age = 17.0 ± 1.0 years	CON vs EXP group differences in physical performance tests: CMJ height = 6.3%; 10 m sprint = 0.2%; 20 m sprint = 0.7% CON vs EXP group differences in 45° COD kinetic variables (side-step cutting): Vertical GRF = 15.3%; propulsive GRF = 7.4%; eccentric impulse = − 5.2%; concentric impulse = 4.2%; total impulse = − 6.8% CON vs EXP group differences in 60° COD kinetic variables (crossover cutting): Vertical GRF = 36.9%; propulsive GRF = 14.0%; eccentric impulse = 15.9%; concentric impulse = 5.4%; total impulse = − 8.5%	The EXP group demonstrated faster sprinting and better jumping performance coupled with improved kinetic parameters during two differing COD tasks
Coutinho et al. [41]	To compare the acute PAPE effects of performing a FIT half-squat in a traditional repetitive and differential learning condition	Participants randomly assigned to 4 different EXP groups: traditional repetitive conditions = (1) 3 sets of 6 repetitions of FIT half-squat, post-test measures taken 30 s after final set; (2) 3 sets of 6 repetitions of FIT half-squat, post-test measures taken 10 min after final set; differential learning conditions (example between-repetition movements include right arm extension, receive tennis ball left hand, right arm abduction) = (3) 3 sets of 6 repetitions of FIT half-squat, post-test measures taken 30 s after final set; (4) 3 sets of 6 repetitions of FIT half-squat, post-test measures taken 10 min after final set No CON group	*n* = 16 male soccer participants, age = 16.2 ± 0.6 years	30 s protocol (condition 1 and 3), repetition vs differential (difference in means): CMJ (cm) = 1.58 (*p* = 0.044); 10 m sprint (s) = 0.01 (*p* = 0.743); 30 m sprint (s) = 0.01 (*p* = 0.837) 10 min (condition 2 and 4), repetition vs differential (difference in means): CMJ (cm) = 1.36 (*p* = 0.038); 10 m sprint (s) = − 0.01 (*p* = 0.641); 30 m sprint (s) = 0.03 (*p* = 0.439)	Despite some individual potentiation responses, there were no potentiation effects found following any of the conditions at the group level
Lloyd et al. [77]	To determine the acute effects of AEL on DJ kinetics	EXP group = 3 drop jumps from 20 cm box holding dumbbells at 15% of body mass (released at the end of eccentric phase to complete concentric phase unloaded) CON group = 3 drop jumps from 20 cm box without dumbbells (body mass only) 10 min rest between protocols	*n* = 15 male soccer participants, age = 16.2 ± 1.01 years	CON vs EXP ES for DJ performance: Jump height, *r* = 0.47 (*p* < 0.05); contact time, *r* = 0.45 (*p* < 0.05); RSI, *g* = − 0.08 (*p* > 0.05); spring-like correlation, *g* = 0.94 (*p* < 0.05); peak COM displacement, *r* = − 0.28 (*p* > 0.05); mean braking power, *g* = − 0.07 (*p* > 0.05); mean propulsive power, *g* = 0.10 (*p* > 0.05); peak landing force, *g* = 0.38 (*p* > 0.05); timing of peak landing force, *r* = 0.10 (*p* > 0.05); braking impulse, *g* = 0.43 (*p* = 0.038); propulsive impulse, *g* = 0.61 (*p* = 0.004); braking duration, *g* = 0.43 (*p* = 0.156); propulsive duration, *r* = 0.519 (*p* = 0.04)	Adolescent athletes can realise a superior jump height during a DJ AEL protocol, but this was achieved alongside longer ground contact times and reduced spring-like behaviour

*AEL* accentuated eccentric loading, *CMJ* countermovement jump, *COD* change of direction, *COM* centre of mass, *CON* control group, *DJ* drop jump, *ERT* eccentric resistance training, *ES* effect size, *EXP* experimental group, *FIT* flywheel inertial training,* g* Hedges’ *g*, *GRF* ground reaction force, *min* minutes, *PAPE* post-activation performance enhancement, *r* Wilcoxon effect size, *RSI* reactive strength index, *s *seconds, *VJ* vertical jump

### Chronic Study Characteristics

Across the 19 chronic studies included for review, there were 538 participants (480 male and 58 female individuals) with a mean age of 15.1 years (range 11.0–18.1 years). Most investigations recruited soccer participants (9 studies; 47.4%), with the remainder including rugby union (2 studies; 10.5%), basketball (2 studies; 10.5%), handball (2 studies; 10.5%), weightlifting (1 study; 5.3%), athletics (1 study; 5.3%), fencing (1 study; 5.3%) and multiple team sport athletes (1 study; 5.3%). A training control group was included in 14 studies (63.3%). The average intervention duration was 7.4 weeks (range 4–12 weeks), with a training frequency of 1.8 sessions per week (range 1–3 sessions). The chosen ERT methods included: NHE (7 studies; 36.8%), FIT (10 studies; 52.6%) and tempo training (2 studies; 10.5%). The study findings for chronic NHE, FIT and tempo training studies can be found in Tables 3, 4 and 5, respectively.

**Table 3:** Chronic Nordic hamstring exercise training studies

Study	Objective	Participants	Training intervention	Relevant findings	Conclusion
Lacome et al. [78]	To compare the effects of HV vs LV eccentric hamstring training on knee-flexor strength and fascicle length	HV: *n* = 10 soccer participants, age = 17.2 ± 0.7 years LV: *n* = 9 soccer participants, age = 17.5 ± 0.7 years	Duration: 2 × 6 week phases (14 weeks total, 2 weeks between phase 1 and 2), 1 session per week EXP group 1 (HV condition): SLDL (4 sets of 6 repetitions), NHE (4 sets of 4 repetitions) EXP group 2 (LV condition): SLDL (1 set of 6 repetitions), NHE (1 set of 4 repetitions) No CON group EXP group continued normal soccer-specific training (approximately 9–10 h per week, 6 training sessions, plus 1–2 games)	Pre- to post-intervention changes after phase 1 (weeks 1–6): Hamstring eccentric strength: HV = 11.4 ± 5.3% (ES = 0.63 ± 0.28), LV = 11.3 ± 7.8% (ES = 1.18 ± 0.77) BF^lh^ FL: HV = 4.8 ± 2.5% (ES = 0.25 ± 0.13), LV = 4.5 ± 5.0% (ES = 0.33 ± 0.35) SM FL: HV = 6.3 ± 6.3% (ES = 0.33 ± 0.32), LV = 4.3 ± 4.7% (ES = 0.39 ± 0.42) Pre- to post-intervention changes after phase 2 (weeks 7–12): Hamstring eccentric strength HV 0.9 ± 7.5% (ES = 0.05 ± 0.43), LV = 1.2 ± 2.9% (ES = 0.12 ± 0.31) BF^lh^ FL: HV = − 0.5 ± 2.0% (ES = − 0.03 ± 0.14), LV = 1.0 ± 2.6% (ES = 0.06 ± 0.15) SM FL: HV = 1.8 ± 4.7% (ES = 0.19 ± 0.52), SM FL LV = −1.6 ± 2.6% (ES = − 0.09 ± 0.15)	LV eccentric training is as effective as HV to improve knee-flexor strength and fascicle length in young elite soccer players
Drury et al. [79]	To investigate the effects of a NHE programme on eccentric hamstring strength	Pre-PHV EXP: *n* = 8 male soccer participants, age = 11.0 ± 0.9 years, MO = −2.8 ± 0.3 years Pre-PHV CON:* n* = 11 male soccer participants, age = 10.9 ± 0.8 years, MO = − 2.7 ± 0.5 years Mid/post-PHV EXP: *n* = 13 male soccer participants, age = 14.0 ± 1.1 years, MO = 0.4 ± 0.9 years Mid/post-PHV CON: *n* = 16 male soccer participants, age = 13.7 ± 1.0 years, MO = 0.1 ± 0.8 years	Duration: 6 weeks, 1–2 sessions per week EXP group NHE progression: 1 session per week of 2 sets of 5 repetitions increasing to 2 sessions per week of 3 sets of 6 repetitions CON group: low-intensity soccer passing drills whilst EXP group completed the above EXP and CON groups continued normal soccer-specific training (volume not specified)	Pre- to post-intervention ESs for relative NHE strength: Pre-PHV EXP = 0.83, pre-PHV CON = − 0.05; mid/post-PHV EXP = 0.53, mid/post-PHV CON = − 0.03 Within-group analyses displayed an increase in relative peak force in both EXP groups, although this was improved to a greater extent in pre-PHV compared with mid/post-PHV (ES = 0.83 vs 0.53) Between-group analyses revealed moderate increases in both maturity groups with the larger effects noted in the pre-PHV group (ES = 1.03 vs 0.87)	A 6 week NHE programme can improve eccentric hamstring strength in male youth soccer players with less mature players achieving mostly greater benefits
Freeman et al. [80]	To compare the effects of NHE and sprint training on eccentric hamstring strength and sprint performance	*n* = 28 male and female multi-sport participants (Australian football, soccer, cricket, baseball and field hockey), age = 16.21 ± 1.34 years	Duration: 4 weeks, 2 sessions per week Eccentric EXP group progression: 2 sessions of 2 sets of 5 repetitions increasing to 2 sessions of 3 sets of 6 repetitions per week (3 min rest between sets, 100% effort on all sets throughout) Sprint EXP group progression: 2 sessions of 6 × 30–40 m increasing to 10 × 30–40 m per week (3 min rest between sets, 100% effort on all sets throughout) No CON group EXP group: aside from training associated with the study, participants completed two additional resistance training and sport-specific technical and tactical sessions per week (details not provided)	Pre- to post-intervention changes in the eccentric EXP group: Eccentric hamstring strength (bilateral) = 9.8% (ES = 0.39; *p* = 0.01) Eccentric hamstring strength (left) = 13.9% (ES = 0.49; *p* = 0.01) Eccentric hamstring strength (right) = 5.7% (ES = 0.24; *p* = 0.10) 0–10 m sprint = 1% (ES = − 0.14; *p* = 0.36) 30–40 m sprint = 0.0% (ES = 0.00; *p* = 0.42) Pre- to post-intervention changes in the sprint EXP group: Eccentric hamstring strength (bilateral) = 6.2% (ES = 0.26; *p* = 0.01) Eccentric hamstring strength (left) = 8.6% (ES = 0.31; *p* = 0.08) Eccentric hamstring strength (right) = 4.0% (ES = 0.16; *p* = 0.01) 0–10 m sprint = 0% (ES = 0; *p* = 0.86) 30–40 m sprint = 8.6% (ES = − 0.83; *p* = 0.10)	The eccentric EXP group improved eccentric hamstring strength but not sprint performance. The sprint EXP group realised improvements in eccentric hamstring strength and sprint performance, namely maximum speed (30–40 m)
Hammami et al. [46]	To investigate the effects of in-season hamstring ERT on sprint and COD performance	Pre-PHV EXP: *n* = 10 male handball participants, age = 11.24 ± 0.93 years Pre-PHV CON: *n* = 10 male handball participants, age = 11.03 ± 0.77 years Post-PHV EXP: *n* = 12 male handball participants, age = 14.00 ± 0.20 years Post-PHV CON: *n* = 13 male handball participants, age = 13.85 ± 0.87 years	Duration: 6 weeks, 2 sessions per week EXP group exercises: glute-ham raise, NHE, single-leg RDL, hip thrust and good morning EXP group progression: 2 sets of 5 repetitions at 60% 1RM increasing to 3 sets of 8 repetitions at 80% 1RM (3–5 s eccentric duration throughout) EXP and CON groups continued normal in-season handball training (5 sessions per week, lasting approximately 90 min each, plus 1 competitive match)	Pre- to post-intervention changes in sprint performance for pre-PHV participants: EXP 10 m time = 5.39% (ES = 0.66; *p* = 0.001), CON 10 m time = − 6.09% (ES = 0.81; *p* = < 0.001) EXP 20 m time = 2.61% (ES = 0.27; *p* = 0.133), CON 20 m time = 0.00% (ES = 0.00; *p* = 0.986) EXP 30 m time EXP = 3.47% (ES = 0.52; *p* = 0.001), CON 30 m time = 2.20% (ES = 0.36; *p* = 0.36) Pre- to post-intervention changes in sprint performance for post-PHV participants: EXP 10 m time = 0.93% (ES = 0.07; *p* = 0.473), CON 10 m time = 6.61% (ES = 0.52; *p* = < 0.001) EXP 20 m time = 2.68% (ES = 0.18; *p* = 0.138), CON 20 m time = − 4.10% (ES = 0.45; *p* = 0.004) EXP 30 m time = 1.94% (ES = 0.23; *p* = 0.050), CON 30 m time = 0.17% (ES = 0.04; *p* = 0.887) Pre- to post-intervention changes in COD performance for pre-PHV participants: EXP 4 × 5 m shuttle run time = 3.23% (ES = 0.67; *p* = 0.001), CON 4 × 5 m shuttle run time = 2.83% (ES = 0.49; *p* = 0.002) EXP T-test time = 4.14% (ES = 0.79; *p* = 0.001), CON T-test time = − 0.13% (ES = 0.02; *p* = 0.957) Pre- to post-intervention changes in COD performance for post-PHV participants: EXP 4 × 5 m shuttle run time = 5.74% (ES = 0.72; *p* = < 0.001), CON 4 × 5 m shuttle run time = 3.99% (ES = 0.54; *p* = 0.002) EXP T-test time = 2.81% (ES = 0.62; *p* = 0.011), CON T-test time = − 16.11% (ES = 2.85; *p* = < 0.001)	An eccentric hamstring strength training programme combined with handball training can improve COD and sprint performance in pre-PHV and post-PHV male participants
Chaabene et al. [48]	To investigate the effects of in-season NHE training on components of physical performance	EXP: *n* = 10 female handball participants, age = 15.9 ± 0.2 years, MO = 3.4 ± 0.4 years CON: *n* = 9 female handball participants, age = 15.9 ± 0.3 years, MO = 3.3 ± 0.5 years	Duration: 8 weeks, 1–3 sessions per week EXP group NHE progression: 1 session per week of 2 sets of 5 repetitions increasing to 3 sessions per week of 3 sets of 8 repetitions EXP and CON group continued normal handball training (5–6 × 60–90 min sessions per week, totalling approximately 8 h)	Pre- to post-intervention ESs: EXP 5 m sprint time = 0.82, CON 5 m sprint time = − 0.46 EXP 10 m sprint time = 0.78, CON 10 m sprint time = − 0.10 EXP 20 m sprint time = 0.68, CON 20 m sprint time = − 0.16 EXP T-test time = 0.74, T-test time CON = − 0.71; CMJ height EXP = 0.85, CON CMJ height = 0.10	The NHE training intervention was effective in improving measures of sprint, COD and jumping performance
Hammami et al. [47]	To examine whether in-season hamstring training would enhance selected performance-related abilities	*n* = 20 male weightlifting participants, age = 11.1 ± 0.8 years MO = − 2.1 ± 0.6 years	Duration: 6 weeks, 2 sessions per week EXP group exercises: glute-ham raise, NHE, single-leg RDL and barbell or dumbbell good morning EXP group progression: 3–5 sets of 10–12 repetitions, 60% of 1RM increasing to 70% of 1RM CON and EXP groups: continued normal weightlifting training routines (5 to 6 × 60–90 min sessions per week) EXP and CON groups continued normal weightlifting programmes (5–6 sessions per week, lasting 60–90 min each)	Pre- to post-intervention changes (mean difference): EXP 1RM (kg) = 35.0 ± 7.4 > 46.7 ± 9.6, CON 1RM (kg) = 33.0 ± 7.5 > 42.1 ± 4.9 EXP SLJ distance (cm) = 169.5 ± 10.3 > 178.5 ± 11.7, CON SLJ distance (cm) = 171.5 ± 12.9 > 167.0 ± 18.5 EXP 3 hop distance (cm) = 440 ± 42.4 > 467 ± 43.9, CON 3 hop distance (cm) = 474 ± 49.1 > 457 ± 51.0 EXP SRL hop distance (cm) = 159.5 ± 10.9 > 164.0 ± 8.1, CON SRL hop distance (cm) = 153.5 ± 15.1 > 156.5 ± 23.4 EXP SLL hop distance (cm) = 152.5 ± 15.5 > 158 ± 9.5, CON SLL hop distance (cm) = 146 ± 21.4 > 148 ± 23.2 EXP agility time (s) = 7.5 ± 0.3 > 7.2 ± 0.3, CON agility time (s) = 0.9 ± 0.5 > 7.9 ± 0.4 EXP 10 m sprint time (s) = 2.1 ± 0.2 > 1.9 ± 0.1, CON 10 m sprint time = 1.9 ± 0.1 > 2.9 ± 0.2 EXP 30 m sprint time (s) = 5.5 ± 0.3 > 5.3 ± 0.3, CON 30-m sprint time (s) = 5.4 ± 0.2 > 5.5 ± 0.3	Eccentric training, undertaken twice weekly for 6 weeks results in positive changes in sprint speed, COD and power performance, but not muscle strength in prepubertal weightlifters
Siddle et al. [43]	To investigate the effects of a LV NHE intervention on functional and structural performance	*n* = 17 elite male academy soccer players, age = 16.65 ± 0.61 years	Duration: 8 weeks, 1–2 sessions per week EXP: NHE EXP progression: 4 sets of 6 repetitions, twice a week for the first 2 weeks. For the remaining 6 weeks, 2 sets of 4 repetitions, once a week No CON group EXP group continued normal soccer-specific training (4 sessions per week, totalling approximately 14 h, plus weekly matches)	Pre- to post-intervention changes (mean difference): IKD PT 60°s^−1^ (N m^−1^) = 9.43, IKD PT 180°s^−1^ (N m^−1^) = 14.72, IKD PT 270°s-^1^ (Nm^−1^) = 6.83; IKD APT 60°s^−1^ (°) = − 1.07, IKD APT 180°s^−1^ (°) = − 1.90, IKD APT 270°s^−1^ (°) = − 3.58 BF^lh^ MT (cm) = − 0.05, SM MT (cm) = 0.13, ST MT (cm) = 0.11, BF^lh^ FL (cm) = 0.21, BF^lh^ MT (°) = − 0.61 Mean COD (s) = − 0.06, 5 m sprint (s) = − 0.01, 10 m sprint (s) = 0.01, 20 m sprint (s) = 0.01	A LV NHE intervention improved COD ability, but not architectural, strength or speed performance in elite youth soccer players

Please note: positive % and ES changes note an improvement in performance; negative % and ES changes note a decline in performance

*APT* angle of peak torque, *BF*^*lh*^ biceps femoris long head, *CMJ* countermovement jump, *COD* change of direction, *CON* control group, *ERT* eccentric resistance training, *ES* effect size, *EXP* experimental group, *FL* fascicle length, *HV* high-volume, *IKD* isokinetic dynamometry, *LV* low-volume, *min* minutes, *MO* maturity offset, *MT* muscle thickness,* n* sample size, *NHE* Nordic hamstring exercise, *Nm* Newton metres, *PA* pennation angle, *PHV* peak-height velocity, *PT* peak torque, *RDL* Romanian deadlift, *s* seconds, *h* hours, *SLDL* stiff-leg deadlift, *SM* semimembranosus, *ST* semitendinosus

**Table 4:** Chronic flywheel inertial training studies

Study	Objective	Participants	Training intervention	Relevant findings	Conclusion
Arede et al. [81]	To compare the effects of standard and inter-repetition variation ERT on measures of physical performance	*n* = 19 female basketball and volleyball participants, age = 15.05 ± 0.5 years, MO = 2.40 ± 0.46 years	Duration: 6 weeks, 2 sessions per week Standard EXP group FIT exercises: backward lunges (1 set of 5 repetitions), defensive-like shuffling steps (1 set of 6 repetitions), side-step (1 set of 5 repetitions) Variable EXP group FIT exercises: same as the standard EXP group, but before each concentric phase, participants were verbally encouraged to perform the movement in one of three directions (1 = 45° right, 2 = 0°, 3 = 45° left), in a randomised order 3 min passive recovery was provided between sets and exercises for both EXP groups No CON group EXP group continued normal team training sessions (3 sessions per week, lasting approximately 90 min, plus 1 competitive match)	Pre- to post-intervention changes: Standard EXP CMJ height = 2.2% (ES = 0.10; *p* = 0.328), variable EXP CMJ height = 0.6% (ES = 0.05; *p* = 0.889) Standard EXP 5 m sprint time = 1.7% (ES = 0.26; *p* = 0.211), variable EXP 5 m sprint time = 1.0% (ES = 0.17; *p* = 0.159) Standard EXP 10 m sprint time = 2.8% (ES = 0.45; *p* = 0.031), variable EXP 10 m sprint time = 1.3% (ES = 0.26; *p* = 0.182) Standard EXP T-test time = 1.1% (ES = 0.20; *p* = 0.108), variable EXP T-test time = 2.7% (ES = 0.51; *p* = 0.050) Standard EXP CMJ height right = 13.6% (ES = 0.47; *p* = 0.045), variable EXP CMJ height right = 10.1% (ES = 0.56; *p* = 0.017) Standard EXP CMJ height left = 16.9% (ES = 0.77; *p* = 0.003), variable EXP CMJ height left = 12.0% (ES = 0.41; *p* = 0.025) Standard EXP HJ distance right = 4.1% (ES = 0.21; *p* = 0.155), variable EXP HJ distance right = 2.6% (ES = 0.31; *p* = 0.292) Standard EXP HJ distance left = 10.0% (ES = 0.45; *p* = 0.036), variable EXP HJ distance left = 3.6% (ES = 0.29; *p* = 0.327) Standard EXP LJ distance right = 13.6% (ES = 0.71; *p* = 0.004), variable EXP LJ distance right = 8.4% (ES = 0.62; *p* = 0.093) Standard EXP LJ distance left = 9.1% (ES = 0.31; *p* = 0.041), variable EXP LJ distance left = 4.9% (ES = 0.42; *p* = 0.093) Standard EXP SLRJ height right = 24.5% (ES = 0.58; *p* = 0.035), variable EXP SLRJ height right = 33.9% (ES = 1.05; *p* = 0.011) Standard EXP SLRJ height left = 16.4% (ES = 0.41; *p* = 0.020), variable EXP SLRJ height left = 12.1% (ES = 0.61; *p* = 0.041)	Standard and variable FIT programmes were found to be beneficial for youth-female team-sport athletes
Fiorilli et al. [89]	To examine the effects of FIT on measures of physical performance when compared to PT	EXP: *n* = 18 male soccer participants, age = 13.21 ± 1.21 years CON: *n* = 16 male soccer participants, age = 13.63 ± 0.80 years	Duration: 6 weeks, 2 sessions per week EXP group FIT exercises: diagonal sprint with cable attached to waist and a simulated soccer shot with cable attached to ankle (maximum concentric effort, resist eccentric phase while back-pedalling for both exercises) EXP group volume: 4 sets of 7 repetitions with 120–180 s rest between sets CON group exercises: PT exercises focusing on improving vertical and horizontal jumping ability CON group volume: 3–4 sets of 4–7 repetitions EXP and CON groups continued normal soccer-specific training (4 sessions per week, lasting approximately 120 min)	Pre- to post-intervention changes (mean difference): FIT EXP DJ height (cm) = 3.34 (*p* = 0.001), CON DJ height (cm) = 0.22 (*p* = NS) FIT EXP DJ CT (s) = 0.10 (*p* = 0.03), CON DJ CT (s) = 0.06 (*p* = NS) FIT EXP DJ RSI (m/s) = − 0.03 (*p* = NS), CON DJ RSI (m/s) = − 0.05 (*p* = NS) FIT EXP 7-HOP average JH (cm) = 3.86 (*p* = 0.003), CON 7-HOP average JH (cm) = 1.56 (*p* = NS) FIT EXP 7-HOP CT (s) = 0.06 (*p* = NS), CON DJ CT (s) = − 0.01 (*p* = NS) FIT EXP 7-HOP RSI (m/s) = − 0.01 (*p* = NS), CON DJ RSI (m/s) = − 0.05 (*p* = NS) FIT EXP SJ height (cm) = 4.13 (*p* = 0.006), CON SJ height (cm) = 3.04 (*p* = 0.008) FIT EXP Illinois agility time (s) = − 3.23 (*p* = < 0.001), CON Illinois agility time (s) = − 0.94 (*p* = 0.03) FIT EXP Y-agility time (s) = − 0.26 (*p* = < 0.001), CON Y-agility time (s) = − 0.08 (*p* = NS) FIT EXP 60 m sprint time (s) = − 0.24 (*p* = < 0.001), CON 60 m sprint time (s) = − 0.07 (*p* = NS)	Isoinertial ERT demonstrated the most beneficial effects in COD performance
Moreno-Azze et al. [82]	To compare the effects of the lateral squat in three different formats from ERT on concentric and eccentric peak and mean power	*n* = 35 male soccer participants, age = 15.4 ± 0.7 years	Duration: 10 weeks, 1 session per week EXP group progression: lateral squat exercise, 2 sets of 6 repetitions increasing to 2 sets of 10 repetitions EXP group conditions: (1) same volume with both legs, beginning with weaker leg (SVW, *n* = 10); (2) same volume with both legs, beginner with stronger leg (SVS, *n* = 14); (3) double volume on weaker leg and beginning with weaker leg (DVW, *n* = 11) No CON group EXP group continued normal soccer-specific training (4 sessions per week, plus 1 competitive match) and a strength/power training session	Pre- to post-intervention ESs for lateral squat: SVW conc mean S = 1.49 (*p* = < 0.01), SVS conc mean S = 1.16 (*p* = < 0.01), DVW conc mean S = 1.07 (*p* = < 0.01) SVW conc mean W = 2.24 (*p* = < 0.01), SVS conc mean W = 1.53 (*p* = < 0.01), DVW conc mean W = 2.04 (*p* = < 0.01) SVW ecc mean S = 1.52 (*p* = < 0.01), SVS ecc mean S = 1.00 (*p* = < 0.01), DVW ecc mean S = 1.17 (*p* = < 0.01) SVW ecc mean W = 1.97 (*p* = < 0.01), SVS ecc mean W = 1.16 (*p* = < 0.01), DVW ecc mean W = 1.08 (*p* = < 0.01) SVW conc peak S = 1.14 (*p* = < 0.01), SVS conc peak S = 1.25 (*p* = < 0.01), DVW conc peak S = 0.92 (*p* = < 0.01) SVW conc peak W = 1.63 (*p* = < 0.01), SVS conc peak W = 1.33 (*p* = < 0.01), DVW conc peak W = 0.77 (*p* = 0.03) SVW ecc peak S = 1.31 (*p* = < 0.01), SVS ecc peak S = 1.08 (*p* = < 0.01), DVW ecc peak S = 1.12 (*p* = < 0.01) SVW ecc peak W = 1.64 (*p* = < 0.01), SVS ecc peak W = 1.19 (*p* = < 0.01), DVW ecc peak W = 1.11 (*p* = < 0.01)	The groups that started with the weaker leg (SVW, DVW) showed greater improvements in the lateral squat test
Di Cagno et al. [40]	To evaluate the effects of FIT on lower limb explosive and reactive strength	EXP: *n* = 26 male fencing participants, age = 17.3 ± 1.9 years CON: *n* = 28 male fencing participants, age = 17.6 ± 2.7 years	Duration: 6 weeks, 1 session per week EXP group FIT exercises: lunge with device behind, lunge with device in front, advance-advance lunge with device behind, advance-advance lunge with device in front EXP group progression: 4 exercises, 7 sets of 3 repetitions increasing to 9 sets of 4 repetitions CON group exercises: drop jumps (50 cm height), jump lunges, squat lunges; circuit training: stiffness jumps with both legs in multi-directions, CMJ onto 50-cm box, hurdle jumps with/without knee bend, speed ladder drills CON group progression: 2 exercises, 7 sets of 1 repetition increasing to 7 sets of 3 repetitions. All sessions finished with a 5 min circuit EXP and CON groups continued normal fencing practice (5 sessions per week, lasting approximately 150 min)	Pre- to post-intervention changes: EXP SJ height (cm) = 3.10, CON SJ height (cm) = 1.67 (*p* = 0.101) EXP CMJ height (cm) = 3.49, CON CMJ height (cm) = 1.18 (*p* = 0.048) EXP 7-RHOP CT (s) = 0.02, CON 7-RHOP CT (s) = 3.24 (*p* = 0.262) EXP 7-RHOP JH (cm) = 6.23, CON 7-RHOP JH (cm) = 3.24 (*p* = 0.095) EXP NHE break-point angle (°) = 2.21, CON NHE break-point angle (°) = 3.36 (*p* = 0.528) Please note: *p*-values presented above are derived from EXP vs CON comparisons Post-intervention to 6 weeks after intervention changes for EXP group: SJ height (cm) = 0.08 (*p* = 0.892), CMJ height (cm) = − 0.57 (*p* = 0.489), 7-RHOP CT (s) = − 0.01 (*p* = 0.257), 7-RHOP JH (cm) = − 3.57 (*p* = 0.000)	The FIT EXP group experienced a significant improvement in lunge and advance-advance lunge amplitude, whilst maintaining the same execution time. Improvements were greater in the FIT EXP group compared with the PE EXP group
de Hoyo et al. [83]	To analyse the effect of an ERT programme on surrogate measures of physical performance	EXP: *n* = 18 male soccer participants, age = 18.0 ± 1.0 years CON: *n* = 15 male soccer participants, age = 17.0 ± 1.0 years	Duration: 10 weeks, 1–2 sessions per week EXP group FIT exercises = prone leg curl and half squat (knee angle = 90°) EXP group progression: 1 session per week of 3 sets of 6 repetitions increasing to 2 sessions per week of 6 sets of 6 repetitions EXP and CON groups continued normal soccer-specific training (4–5 × 60–90 min sessions per week, plus 1 match)	Pre- to post-intervention changes: EXP CMJ height = 7.6% (ES = 0.58), CON CMJ height = − 1.7% (ES = − 0.18) EXP 10 m sprint time = 1.0% (ES = 0.15), CON 10 m sprint time = − 0.3% (ES = − 0.05) EXP 20 m sprint time = 1.5% (ES = 0.32), CON 20 m sprint time = − 0.1% (ES = − 0.03) EXP 10 m flying sprint time = 3.3% (ES = 0.95), CON 10 m flying sprint time = 0.2% (ES = 0.05)	The EXP group demonstrated greater improvements in surrogate measures of physical performance compared with the CON group
de Hoyo et al. [84]	To analyse the effects of ERT on kinetic parameters during COD performance	*n* = 31 male soccer participants, age = 17.0 ± 1.0 years	Duration: 10 weeks, 1–2 sessions per week EXP group FIT exercises = prone leg curl and half squat (knee angle = 90°) EXP group progression: 1 session per week of 3 sets of 6 repetitions increasing to 2 sessions per week of 6 sets of 6 repetitions EXP and CON groups continued normal soccer-specific training (4–5 × 60–90 min sessions per week, plus 1 match)	Pre- to post-intervention changes for crossover cutting at 60°: EXP CT = 9.1% (ES = 0.75), CON CT = 1.3% (ES = 0.09) EXP BT = 10.0% (ES = 0.35), CON BT = 3.3% (ES = 0.15) EXP PT = 4.2% (ES = 0.13), CON PT = 1.5% (ES = 0.09) EXP rPB force = 26.1% (ES = 0.75), CON rPB force = 31.5% (ES = 0.08) EXP rPF = 26.4% (ES = 1.34), CON rPF = 2.1% (ES = 0.07) EXP rTOT_IMP = 14.6% (ES = 0.61), CON rTOT_IMP = 3.1% (ES = 0.10) EXP rB_IMP = 22.4% (ES = 0.76), CON rB_IMP = 5.7% (ES = 0.16) EXP rP_IMP = 14.7% (ES = 0.46), CON rP_IMP = − 0.1% (ES = 0.00) Pre- to post-intervention changes for side-step cutting at 45°: EXP CT = 17.7% (ES = 1.19), CON CT = 1.4% (ES = 0.08) EXP BT = 22.6% (ES = 1.24), CON BT = 6.5% (ES = 0.32) EXP PT = 13.6% (ES = 0.70), CON PT = − 0.5% (ES = − 0.03) EXP rPB force = 31.5% (ES = 0.75), CON rPB force = 8.0% (ES = 0.27) EXP rPF = 13.8% (ES = 0.68), CON rPF = 7.3% (ES = 0.23) EXP rTOT_IMP = 12.3% (ES = 0.48), CON rTOT_IMP = 2.2% (ES = 0.07) EXP rB_IMP = 14.8% (ES = 0.50), CON rB_IMP = − 1.7% (ES = − 0.06) EXP rP_IMP = 8.9% (ES = 0.26), CON rP_IMP = − 14.0% (ES = − 0.31)	ERT led to greater braking and propulsive forces and impulses, and a lower braking and propulsive contact time during COD tasks
Stojanović et al. [48]	To compare the effects of FIT and traditional strength training on fitness attributes	EXP 1: *n* = 12 male basketball participants, age = 17.58 ± 0.52 years EXP 2: *n* = 12 male basketball participants, age = 17.52 ± 0.58 years CON: *n* = 12 male basketball participants, age = 17.56 ± 0.54 years	Duration: 8 weeks, 1–2 sessions per week EXP group 1: FIT EXP group 2: free weight training EXP groups 1 and 2 exercises: one-arm dumbbell row, rotational Paloff press, biceps curl + upright row, half squat and RDL EXP groups 1 and 2 progression: 1 sessions per week of 2 sets of 8 to 15 repetitions increasing to 2 sessions per week of 4 sets of 8 to 15 repetitions EXP and CON groups continued normal basketball training (5 sessions per week, lasting 90 min) and weekend games plus one 25–30 min bodyweight strength session per week (volume not specified)	Pre- to post-intervention changes: EXP 1 ISOMET = 18.7% (ES = 1.88), EXP 2 ISOMET = 16.6% (ES = 1.52), CON ISOMET = 2.9% (ES = 0.51) EXP 1 CMJ = 11.7% (ES = 2.19), EXP 2 CMJ = 6.8% (ES = 1.12), CON CMJ = 0.3% (ES = 0.05) EXP 1 5 m sprint = 10.3% (ES = 3.79), EXP 2 5 m sprints = 5.9% (ES = 1.15), CON 5 m sprint = 3.4% (ES = 0.84) EXP 1 20 m sprint = 4.1% (ES = 1.29), EXP 2 20 m sprints = 3.4% (ES = 1.04), CON 20 m sprint = 0.6% (ES = 0.05) EXP 1 *t*-test = 2.4% (ES = 2.78), EXP 2 *t*-tests = 1.4% (ES = 1.64), CON *t*-test = 0.6% (ES = 0.92)	Eight weeks of FIT induces superior improvements in CMJ, 5-m sprint time and COD ability, but not isometric strength compared with volume matched traditional strength training
Westblad et al. [39]	To compare the effects of autoregulated FIT with traditional strength training on measures of physical performance	EXP: *n* = 14 male and female athletics participants, MO = − 0.8 ± 1.6 years CON: *n* = 11 male and female athletics participants, MO = − 0.8 ± 1.5 years Combined age = 11.8 ± 0.9 years	Duration: 6 weeks, 2 sessions per week EXP group: FIT squat CON group: free-weight barbell squat EXP and CON progression: 4 sets of 6 repetitions. Intensity controlled via autoregulation: self-reported RPE, coaches set a load to match a set-RPE of 8 CON group increased intensity via adding mass, EXP group increased intensity via adjusting inertia or increasing/decreasing movement speed EXP and CON groups continued normal track and field training (1–3 sessions per week, including a mixture of sprinting hurdles, long jumps and shot put)	Pre- to post-intervention changes for EXP and CON: 10-m acceleration ^a^*p* = 0.08, ^b^*p* = 0.45, ^c^*p* = 0.60 20 m flying sprint ^a^*p* = 0.94, ^b^*p* = 0.11, ^c^*p* = 0.53 30 m sprint ^a^*p* = 0.36, ^b^*p* = 0.13, ^c^*p* = 0.88 SJ ^a^*p* = 0.01, ^b^*p* = 0.65, ^c^*p* = 0.48 CMJ ^a^*p* = 0.40, ^b^*p* = 0.47, ^c^*p* = 0.77 Please note: ^a^main effect: time; ^b^main effect: group; ^c^interactive effect: group × time	EXP and CON improved jumping but not running performance
Murton et al. [85]	To investigate the effects of FIT vs traditional resistance training	*n* = 16 elite male academy rugby union participants, age = 18.0 ± 1.0 years	Duration: 4 weeks, 2 sessions per week EXP group exercises: FIT squat, RDL and Bulgarian split squat CON group exercises: free-weight squat, RDL and Bulgarian split squat EXP and CON group progression: 4 sets of 6 repetitions increasing to 5 sets of 8 repetitions EXP and CON groups continued normal rugby-specific training (volume not specified)	Pre- to post-intervention changes: EXP CMJ-PP (W·kg^−1^) = 1.96 (ES = 0.55), CON CMJ-PP (W·kg^−1^) = 2.26 (ES = 0.39) EXP CMJ-PF (N·kg^−1^) = 0.45 (ES = 0.18), CON CMJ-PF (N·kg^−1^) = 0.93 (ES = 0.42) EXP CMJ height (cm) = 1.81 (ES = 0.43), CON CMJ height (cm) = 2.79 (ES = 0.51) EXP SJ-PP (W·kg-^1^) = 4.27 (ES = 0.22), CON SJ-PP (W·kg^−1^) = − 0.29 (ES = − 0.05) EXP SJ-PF (N·kg^−1^) = 0.42 (ES = 0.27), CON SJ-PF (N·kg^−1^) = 0.50 (ES = 0.27) EXP SJ height (cm) = 1.65 (ES = 0.47), CON SJ height (cm) = 3.68 (ES = 0.88) EXP RSI = 0.13 (ES = 0.30), CON RSI = 0.25 (ES = 0.57) No significant between-group differences	EXP and CON were found to be effective for developing lower-body strength and power qualities
Raya-González et al. [86]	To investigate the effects of FIT on physical performance	*n* = 20 under-16 age group male soccer participants	Duration: 10 weeks, 1 session per week EXP group exercise: FIT lateral squat EXP group progression: 2 sets of 8 repetitions increasing to 4 sets of 8 repetitions in weeks 7 to 8 before decreasing to 3 sets of 8 repetitions and 2 sets of 8 repetitions in weeks 9 and 10, respectively EXP and CON groups continued normal soccer-specific training (volume not specified)	Pre- to post-intervention changes in jump and sprint performance: EXP CMJ_D_ (cm) = 2.70 ± 0.77 (ES = 0.75; *p* = 0.01), CON CMJ_D_ (cm) = 0.21 ± 0.94 (ES = 0.12; *p* = 0.50) EXP CMJ_ND_ (cm) = 4.18 ± 0.69 (ES = 1.28; *p* = 0.001), CON CMJ_ND_ (cm) = − 0.11 ± 0.31 (ES = − 0.03; *p* = 0.29) EXP 10 m sprint (s) = 0.05 ± 0.11 (ES = 0.73; *p* = 0.18), CON 10 m sprint (s) = − 0.02 ± 0.09 (ES = − 0.15; *p* = 0.61) EXP 20 m sprint (s) = 0.04 ± 0.11 (ES = 0.54; *p* = 0.31), CON 20 m sprint (s) = 0.00 ± 0.10 (ES = 0.00; *p* = 1.00) EXP 30 m sprint (s) = 0.05 ± 0.14 (ES = 0.52; *p* = 0.34), CON 30 m sprint (s) = − 0.02 ± 0.10 (ES = − 0.13; *p* = 0.57) Pre- to post-intervention changes in COD performance: EXP COD10_D_ (s) = 0.23 ± 0.09 (ES = 1.95; *p* = 0.001), COD10_D_ (s) = 0.12 ± 0.08 (ES = 1.30; *p* = 0.01) EXP COD10_ND_ (s) = 0.20 ± 0.13 (ES = 1.26; *p* = 0.003), COD10_ND_ (s) = − 0.01 ± 0.02 (ES = − 0.03; *p* = 0.68) EXP COD_def_10_D_ (s) = 0.18 ± 0.12 (ES = 1.42; *p* = 0.001), COD_def_10_D_ (s) = 0.03 ± 0.04 (ES = 0.26; *p* = 0.03) EXP COD_def_10_ND_ (s) = 0.15 ± 0.13 (ES = 1.07; *p* = 0.007), COD_def_10_ND_ (s) = 0.01 ± 0.09 (ES = 0.08; *p* = 0.69) EXP COD20_D_ (s) = 0.19 ± 0.09 (ES = 1.40; *p* = 0.04), COD20_D_ (s) = − 0.01 ± 0.04 (ES = 0.20; *p* = 0.45) EXP COD20_ND_ (s) = 0.28 ± 0.08 (ES = 2.20; *p* = 0.03), COD20_ND_ (s) = − 0.02 ± 0.12 (ES = − 0.12; *p* = 0.68) EXP COD_def_20_D_ (s) = 0.16 ± 0.18 (ES = 0.97; *p* = 0.02), COD_def_20_D_ (s) = 0.01 ± 0.14 (ES = 0.05; *p* = 0.91) EXP COD_def_20_ND_ (s) = 0.24 ± 0.13 (ES = 2.02; *p* = 0.03), COD_def_20_ND_ (s) = 0.01 ± 0.18 (ES = 0.05; *p* = 0.90)	FIT is suitable for improving jumping and COD abilities

Please note: positive % and ES changes note an improvement in performance; negative % and ES changes note a decline in performance

*7-RHOP* 7 repeated hopping, *ASI* asymmetry index, *BT* braking time, *CMJ* countermovement jump, *COD10* change of direction over 5 + 5 m with a 90° turn, *COD*_*def*_*10* change of direction deficit over 5 + 5 m with a 90° turn, *COD20* change of direction over 10 + 10 m with a 90° turn, *COD*_*def*_*20* change of direction deficit over 10 + 10 m with a 90° turn, *CON* control group, conc concentric, *CT* contact time, _*D*_ dominant leg, *DJ* drop jump, *ecc* eccentric, *ERT* eccentric resistance training, *EXP* experimental group, *FIT* flywheel inertial training, *HJ* horizontal jump, *ISOMET* isometric strength test, *JH* jump height, *LJ* lateral jump, *min* minutes, *MO* maturity offset, _*ND*_ non-dominant leg, *NS* non-significant, *PF* relative peak force, *PP* relative peak power, *PE* plyometric exercise, *PT* propulsive time, *RDL* Romanian deadlift, *rPB* relative peak braking, *RPE* rate of perceived exertion, *rPF* relative propulsive force, *rB_IMP* relative braking impulse, *rP_IMP* relative propulsive impulse, *s* seconds, *S* stronger leg, *RSI* reactive strength index, *rTOT_IMP* relative total impulse, *SJ* squat jump, *SLRJ* diagonal single-leg rebound jump, *W* weaker leg

**Table 5:** Chronic eccentric resistance training studies

Study	Objective	Participants	Training intervention	Relevant findings	Conclusion
Bourgeois et al. [87]	To investigate the effects of eccentric phase-emphasis strength training on unilateral strength and 180° and 45° COD performance	EXP: *n* = 12 male rugby union participants, age = 15.0 ± 0.9 years CON: *n* = 6 male rugby union participants, age = 15.3 ± 0.5 years	Duration: 6 weeks, 3 sessions per week EXP group: weeks 1–6 = upper and lower body isoinertial resistance exercises with controlled, 3-s eccentric durations, followed by a concentric action performed as “fast as possible” CON group: weeks 15–20 = same exercises, sets and repetitions but with no constraints on tempo EXP and CON group progression: 3 sets of 8–10 repetitions increasing to 3 sets of 4–10 repetitions EXP and CON group main exercises: lower body = parallel back squat, hexagon-bar squat; upper body = flat bench press, standing overhead press Performance was assessed before (pre-test) and after completion of a 6 week intervention (post-test_1_) and 3 weeks post-cessation (post-test_2_) Participants were further split into FAST and SLOW groups based upon a median split of baseline COD time assessments	Pre-test, post-test_1_, post-test_2_ changes for right leg isometric peak force (N/N): EXP FAST = 1.72 ± 0.18, 1.85 ± 0.29, 2.14 ± 0.25 EXP SLOW = 1.79 ± 0.30, 1.67 ± 0.24, 2.14 ± 0.25 CON FAST = 1.66 ± 0.24, 2.08 ± 0.36, 1.77 ± 0.34 CON SLOW = 1.50 ± 0.06, 1.95 ± 0.30, 1.78 ± 0.03 Pre-test, post-test_1_, post-test_2_ changes for right leg 180° approach time (s): EXP FAST = 1.09 ± 0.08, 1.08 ± 0.04, 1.09 ± 0.06 EXP SLOW = 1.09 ± 0.02, 1.10 ± 0.04, 1.11 ± 0.02 CON FAST = 1.09 ± 0.02, 1.12 ± 0.07, 1.13 ± 0.05 CON SLOW = 1.13 ± 0.04, 1.07 ± 0.07, 1.12 ± 0.02 Pre-test, post-test_1_, post-test_2_ changes for right leg 180° exit time (s): EXP FAST = 1.14 ± 0.14, 1.11 ± 0.08, 1.07 ± 0.04 EXP SLOW = 1.04 ± 0.06, 1.11 ± 0.08, 1.10 ± 0.08 CON FAST = 1.07 ± 0.07, 1.09 ± 0.04, 0.75 ± 0.03 CON SLOW = 1.14 ± 0.07, 1.04 ± 0.02, 0.74 ± 0.12 Pre-test, post-test_1_, post-test_2_ changes for right leg 180° total time (s): EXP FAST = 3.00 ± 0.18, 2.98 ± 0.08, 2.90 ± 0.08 EXP SLOW = 3.16 ± 0.12, 3.01 ± 0.10, 2.99 ± 0.17 CON FAST = 3.03 ± 0.15, 2.94 ± 0.06, 2.94 ± 0.04 CON SLOW = 2.90 ± 0.16, 2.98 ± 0.23, 2.94 ± 0.19 Pre-test, post-test_1_, post-test_2_ changes for right leg 45° approach time (s): EXP FAST = 0.91 ± 0.03, 0.96 ± 0.02, 0.95 ± 0.01 EXP SLOW = 0.99 ± 0.03, 1.00 ± 0.06, 1.00 ± 0.04 CON FAST = 0.99 ± 0.06, 1.02 ± 0.06, 1.13 ± 0.05 CON SLOW = 0.96 ± 0.03, 0.97 ± 0.06, 1.12 ± 0.02 Pre-test, post-test_1_, post-test_2_ changes for right leg 45° exit time (s): EXP FAST = 0.63 ± 0.03, 0.69 ± 0.06, 0.64 ± 0.02 EXP SLOW = 0.70 ± 0.04, 0.73 ± 0.05, 0.73 ± 0.05 CON FAST = 1.07 ± 0.07, 0.77 ± 0.05, 0.75 ± 0.03 CON SLOW = 1.14 ± 0.07, 0.65 ± 0.04, 0.74 ± 0.12 Pre-test, post-test_1_, post-test_2_ changes for right leg 45° total time (s): EXP FAST = 1.54 ± 0.04, 1.65 ± 0.07, 1.60 ± 0.01 EXP SLOW = 1.70 ± 0.07, 1.74 ± 0.11, 1.73 ± 0.09 CON FAST = 1.65 ± 0.13, 1.66 ± 0.09, 1.71 ± 0.14 CON SLOW = 1.71 ± 0.06, 1.82 ± 0.09, 1.76 ± 0.12	CON was more beneficial in facilitating enhancements in unilateral isometric strength at post-test_1_, while EXP was more effective in retaining and further enhancing unilateral isometric strength CON was more beneficial for approach and exit times in both 180° and 45° COD tasks, while EXP was more beneficial for total times in 180° COD tasks. Finally, SLOW benefitted more from EXP, while FAST experienced meaningful improvements of lesser magnitudes
Dafkou et al. [88]	To investigate the effects of eccentric, balance and core exercises on neuromuscular adaptations	EXP: *n* = 11 male soccer participants, age = 17.7 ± 1.15 years CON:* n* = 10 male soccer participants, age = 18.1 ± 0.57 years	Duration: 8 weeks, 2 sessions per week EXP group: sliding single leg curl (6–8 s eccentric tempo), 5 single-leg balance variations and 4 core-muscle exercises EXP group progression: 2 sets of 6 repetitions increasing to 4 sets of 10 repetitions, 1–2 min rest between sets EXP and CON groups continued normal soccer specific training (4–5 training sessions, plus one game per week)	Hamstring concentric peak torque changes: EXP 240°/s DL = 5.09 ± 7.38; CON 240°/s DL = − 1.56 ± 34.97 EXP 180°/s DL = 1.99 ± 6.20; CON 180°/s DL = − 5.51 ± 33.68 EXP 30°/s DL = − 2.72 ± 14.95; CON 30°/s DL = − 8.38 ± 10.24 Hamstring eccentric peak torque changes: EXP 240°/s DL = 6.37 ± 30.18; CON 240°/s DL = − 0.42 ± 1.79 EXP 180°/s DL = 4.82 ± 25.81; CON 180°/s DL = − 1.96 ± 4.48 EXP 30°/s DL = 5.14 ± 12.99 CON 30°/s DL = − 1.39 ± 23.22 Quadricep concentric peak torque changes: EXP 240°/s DL = 13.32 ± 28.52; CON 240°/s DL = − 3.99 ± 32.66 EXP 180°/s DL = 8.84 ± 6.24; CON 180°/s DL = − 1.53 ± 14.3 EXP 30°/s DL = − 1.28 ± 21.05; CON 30°/s DL = − 3.85 ± 25.65 Quadricep eccentric peak torque changes: EXP 240°/s DL = 10.97 ± 93.89; CON 240°/s DL = 3.59 ± 16.15 EXP 180°/s DL = 4.64 ± 28.98; CON 180°/s DL = 1.03 ± 16.25 EXP 30°/s DL = 1.08 ± 29.78 CON 30°/s DL = 1.20 ± 53.53	Incorporating small doses of hamstring eccentric, proprioception and core stability exercises into a youth soccer training programme improves eccentric and concentric strength for the quadriceps and hamstrings

*COD* change of direction, *CON* control group, *DL* dominant limb, *ES* effect size, *EXP* experimental group, *n* sample size, *NDL* non-dominant limb, *RM* repetition maximum, *s* seconds, *SLJ* standing long jump, *SLL* single left leg hop, *SRL* single right leg hop

### Risk of Bias Assessment

Following the Downs and Black assessment, 3 studies (23.1%) were excluded after reporting a high risk of bias (study quality percentage score < 50.0%) [66–68]. Of the 3 acute and 19 chronic studies, 8 studies (27.3%) displayed a low risk of bias, and the remaining 14 studies (72.7%) scored a moderate risk of bias. Please refer to Table 6 for a full list of studies and their respective modified Downs and Black checklist scores.

**Table 6:** Modified Downs and Black checklist results

Study	Downs and Black checklist question number																									
	Reporting									External validity		Internal validity, bias				Internal validity, selection bias								Power		Rating
Acute studies	1	2	3	4	5	6	7	8	9	10	11	12	13	14	15	16	17	18	19	20	21	22	23	24	25	
de Hoyo et al. [76]	1	1	1	1	1	1	1	0	0	1	1	0	1	0	0	0	1	0	0	0	0	1	1	1	0	Fair
Coutinho et al. [41]	1	1	1	1	1	1	1	0	1	1	1	0	1	1	1	1	1	0	1	0	0	0	0	0	0	Fair
Lloyd et al. [77]	1	1	1	1	2	1	1	0	0	1	1	0	1	1	1	1	1	1	1	1	0	1	1	1	0	High

Chronic studies	1	2	3	4	5	6	7	8	9	10	11	12	13	14	15	16	17	18	19	20	21	22	23	24	25	Rating
Lacome et al. [78]	1	1	1	1	1	1	1	0	0	1	1	0	0	0	1	1	1	1	0	0	0	1	0	0	0	Fair
Drury et al. [79]	1	1	1	1	1	1	1	1	0	1	1	0	0	1	1	1	1	1	1	1	0	1	0	1	0	High
Hammami et al. [46]	1	1	0	1	1	1	1	1	1	1	1	1	1	0	0	0	1	0	0	0	0	1	0	1	0	Fair
Chaabene et al. [48]	1	1	1	1	1	1	1	1	0	1	1	0	1	1	0	0	0	0	0	0	0	1	0	1	0	Fair
Freeman et al. [80]	1	1	1	1	2	1	1	1	1	1	1	0	0	1	1	1	1	1	1	1	0	1	0	1	0	High
Siddle et al. [43]	1	1	1	1	2	1	1	1	1	1	1	0	0	1	1	1	1	1	1	1	0	1	0	1	0	High
Arede et al. [81]	1	1	1	1	1	1	1	1	1	1	1	0	1	1	1	1	1	0	0	0	0	1	0	0	0	High
Fiorilli et al. [40]	1	1	1	1	2	1	1	1	1	1	1	0	1	0	1	1	1	1	0	1	0	1	1	0	0	High
Moreno-Azze et al. [82]	1	1	1	1	1	1	1	1	1	1	1	0	1	1	1	0	1	0	1	1	0	1	0	1	0	High
Di Cagno et al. [40]	1	1	1	1	2	1	1	0	1	1	1	0	1	0	1	0	1	1	0	0	0	1	0	0	0	Fair
de Hoyo et al. [83]	1	1	1	1	1	1	1	1	0	1	1	1	0	0	0	0	1	0	0	0	0	1	0	1	0	Fair
de Hoyo et al. [84]	1	1	1	1	1	1	1	1	1	1	1	1	1	0	0	0	1	0	0	0	0	1	0	0	0	Fair
Stojanovic et al. [48]	1	1	1	1	1	1	1	1	1	1	1	1	1	0	0	1	1	0	0	0	0	1	1	0	0	High
Westblad et al. [39]	1	1	1	1	1	1	1	1	0	1	1	1	1	0	0	0	1	0	0	0	0	1	0	1	0	Fair
Murton et al. [85]	1	1	1	1	1	1	1	0	0	1	1	0	1	0	0	1	1	0	0	0	0	1	1	1	0	Fair
Raya-González et al. [86]	1	1	1	1	1	1	1	0	0	1	1	1	1	0	0	0	1	0	0	1	0	1	1	1	0	Fair
Bourgeois et al. [87]	1	1	1	1	1	1	1	1	0	1	1	0	0	0	0	1	1	1	0	0	0	1	0	1	0	Fair
Dafkou et al. [88]	1	1	1	1	1	1	1	0	0	1	1	1	1	0	0	1	1	0	0	0	0	1	1	1	0	Fair
Hammami et al. [47]	1	1	1	1	1	1	1	0	1	1	1	1	1	0	0	0	1	0	0	0	0	1	1	1	0	Fair

2 = yes; 1 = yes/partially (question 5); 0 = no/unable to determine

### Acute Effects of ERT on Measures of Physical Performance

The current understanding of acute responses to ERT in youth populations is limited. Six acute ERT studies were retrieved during the search process, with only 3 meeting the necessary inclusion criteria (Table 2), making it difficult to draw meaningful conclusions. In these investigations, FIT and AEL were the chosen methods [44, 69, 70], with the aim of establishing a post-activation performance enhancement (PAPE) effect or simply observing the differences in kinetic variables. Participants in these studies had a mean ± standard deviation age of 16.5 ± 0.5 years (range 16.2–17.0 years).

#### PAPE Following FIT

Post-activation performance enhancement features a high-intensity conditioning contraction(s) and is designed to increase voluntary muscular performance during a subsequent exercise test [71, 72]. The acute FIT studies that integrated a PAPE design included male soccer participants with a variety of physical performance tests evaluated pre- and post-intervention [44, 69]. de Hoyo et al. [69] used a half-squat and found greater improvements in countermovement jump (CMJ) height and 10 and 20 m sprint times, in comparison with a control group, who underwent 5 min of stationary cycling. The experimental group also experienced superior results for several kinetic parameters during 45 and 60 degree COD tasks (Table 2). Coutinho et al. [44] implemented a similar protocol, whereby participants were randomly assigned to 4 different experimental conditions. These comprised a half-squat using traditional repetitive and differential learning styles [73], with performance being assessed in the CMJ, sprint and repeated COD tasks pre-intervention and at 30 s and 10 min post-intervention. The differential learning approach included random movement perturbations (i.e. right arm overhead, left arm abduction, receive tennis ball left hand) that were used to challenge participants in a different way to traditional linear repetitive methods [74]. Despite all conditions resulting in performance decrements, this was comparatively less following the differential learning protocols (please refer to Table 2), which is in accordance with similar investigations [73, 75, 76]. The authors suggested that the lack of positive findings was as a result of the participants’ limited FIT experience, making them more susceptible to fatigue and less able to realise a performance enhancement [71, 77]. However, it is also possible that the 30 s post-intervention rest period was insufficient to enable fatigue to dissipate, whereas the 10 min rest period was too long, resulting in a loss of potentiation [77–79]. Equally, all participants also underwent testing conditions 3 min pre-intervention, which amounted to a large volume of performance assessments and training in a small period of time. While de Hoyo et al. [69] implemented 2 familiarisation sessions, Coutinho et al. [44] did not report the inclusion of any familiarisation trials. Given the relatively unique mechanical demands of FIT, prior studies have recommended a minimum of 3 familiarisation trials for participants to become acquainted [48, 80, 81], which is likely even more important when working with children and adolescents [82, 83]. Cumulatively, these reasons explain the differences in results from those of de Hoyo et al. [69] and suggest that careful consideration regarding familiarisation prior to implementing FIT is crucial [48, 84].

The rationale for utilising ERT as a form of PAPE relates to the underlying mechanisms [71]. Eccentric muscle actions, in comparison with concentric muscle actions, are believed to lead to a preferential recruitment of higher order motor units because of a higher motor unit discharge rate and synchrony [39, 85–88]. The potential training effects resulting from ERT may also favour adaptations in faster eccentric-concentric coupled exercises that utilise the stretch–shortening cycle (SSC), such as jumping, sprinting and COD tasks [89–91]. The majority of current acute ERT studies in youth focus on FIT, probably because of its portability making it an easy-to-use training tool in S&C practice [92]. However, there are numerous other ERT strategies that merit inclusion in research and practice as a PAPE protocol amongst youth athletes, including tempo training [42, 93, 94], AEL [5, 7, 39], accelerated eccentrics and accelerated eccentric loading [95]. It is important that further investigation is undertaken to better understand the acute effects following ERT in youth athletes. Additional studies aiming to identify the most effective training volume, intensity and recovery period following FIT in a PAPE complex are also necessary before informative guidelines can be provided.

#### Kinetic Analysis of AEL

AEL is sometimes thought to be a supramaximal exercise only whereby the eccentric load is prescribed in excess of the concentric load and is implemented using equipment such as weight releasers [5]. Alternatively, during submaximal AEL, the eccentric load does not exceed the concentric 1-repetition maximum and is favoured in situations where adaptations in SSC performance are desirable [5, 96]. The current review found improvements in drop jump (DJ) performance with the addition of AEL [70]. Specifically, Lloyd et al. [70] compared the kinetics of a dumbbell AEL DJ at 15% of body mass with an unloaded DJ (Table 2). The results suggest that dumbbell AEL can be used to realise a superior DJ height; however, this is at the expense of longer ground contact times and reduced spring-like behaviour that may limit the carry over to faster SSC activities such as sprinting [97], instead favouring COD tasks of greater angles [98, 99]. Significant increases in braking and propulsive impulse were observed in the AEL condition owing to the extended time spent on the ground facilitating a longer period to generate force [100]. The AEL condition also experienced a higher peak centre of mass displacement (jump height), supporting the belief that greater jump heights are realised with an increase in displacement and movement time prior to take-off as a result of a higher active state and a greater fraction of actin-binding sites for cross-bridge formation [101–106]. Although not quantified, this could also have resulted in a greater velocity during the eccentric phase, which would be particularly beneficial for stimulating a greater SSC response and higher force outputs at the beginning of the concentric phase [101–103]. Cumulatively, these results suggest that dumbbell AEL can be used in youth athletes to acutely increase DJ height; however, careful consideration of the desired movement strategy and participant strength levels is also necessary. For example, with the addition of dumbbells at 15% of body mass, longer ground contact times and reduced spring-like behaviour are apparent, likely because of the need to undergo a greater countermovement prior to take-off. Therefore, if S&C coaches wish to develop fast SSC ability, unloaded DJ methods may be favourable.

An assessment of strength may have also facilitated a more thorough understanding of the optimal dumbbell mass and drop height. Despite incorporating a standardised box height of 30 cm [70], previous research has recommended the incorporation of an optimal drop height based upon the highest individual reactive strength index (RSI) values [107]. Once an RSI assessment has been carried out, a comparison of the responses between 2 groups based upon a median split of RSI values could have provided further insight. Similarly, a strength assessment would help to comprehend those who respond better to AEL methods, which in turn would facilitate the development of more targeted strength and power interventions. The use of 3-dimensional motion capture would also enable a more thorough understanding of the kinematic differences between AEL and unloaded DJs (i.e. hip and knee joint flexion angles), as well as an accurate quantification of the time point at which the dumbbells are released. Lastly, insight into the adaptations following chronic exposure to dumbbell AEL in youth athletes across maturity groups is also warranted as the longer ground contact times observed may be alleviated based upon the physical changes that take place during growth and maturation (i.e. increased muscle mass, strength, power, SSC capability) [18, 108, 109].

Independent of sex age and maturity status, previous investigations have consistently found children and adolescents perform better in the CMJ when compared to DJs that range from drop heights of 10–70 cm [110–112]. In contrast, comparable studies in adults have reported increases in performance from squat jumps (SJs) to CMJs and CMJs to DJs (5–42% and 3–32%, respectively) [103–106, 113, 114]. It appears that children and adolescents, unlike adults, are unable to utilise the greater eccentric pre-loading forces to increase jump performance beyond that of a CMJ. The mechanisms underlying these differences between children and adults are suggested to be greater elastic energy utilisation, augmentations in the stretch reflex, differences in fibre-type composition and neural maturation, all of which improve as children experience growth and maturation, contributing to enhanced SSC performance [18, 108, 109, 115, 116]. The combination of dumbbells at 15% of body mass and a DJ from 30 cm may have been too intense for youth athletes to realise a performance enhancement. Future research should therefore replicate the work of Lloyd et al. [70], but instead implement a CMJ, rather than a DJ to provide another option for S&C coaches working with youth athletes. Further investigation incorporating a range of drop heights and dumbbell loads in older adolescents (i.e. 18 years of age or under) is also necessary. For more thorough comparisons between SJs, CMJs and DJs to be made in youth athletes, research must also build upon current protocols and include additional kinetic and kinematic variables in addition to jump height and RSI (i.e. braking and propulsion phase time, force, velocity and countermovement displacement). This would facilitate a greater understanding of the changes between jumps and across populations.

### Chronic Effects of NHE on Physical Performance

The inclusion of the NHE in S&C programming is currently supported across a number of youth sports [51, 52, 117, 118]. Benefits have been observed in children as young as 10 years of age [119], with a wealth of research suggesting the NHE helps reduce the risk factors associated with HSI [117, 120–123]. For example, 2 separate systematic reviews by van Dyk et al. [122] and Al Attar et al. [124] found programmes including the NHE reduced hamstring injuries by up to 51% across multiple sports and in athletes aged between 13 and 40 years. In further support of this, Drury et al. [33] recommended that the NHE is consistently integrated throughout all stages of a child’s growth and maturation with an emphasis upon increasing training volume up to the age at PHV, before increasing intensity thereafter. The rationale behind this is to support the natural developments in FL that occur primarily during the pre-PHV period, and the subsequent increase in muscle cross-sectional area that occurs during and after the PHV period [125, 126]. The current review notes the positive effects on measures of physical performance, including eccentric hamstring strength, jump, sprint speed and COD performance following the NHE [57, 59, 127, 128] (Table 3). Participants in these studies had a mean ± standard deviation age of 14.2 ± 3.0 years (range 11.0–17.5 years).

#### Eccentric Hamstring Strength and Muscle Architecture

This review identified 4 studies that evaluated the effects of the NHE on eccentric hamstring strength [46, 119, 127, 128], with 2 of these including measurements of muscle architecture [128, 129] (Table 3). In support of a recent systematic review by Cuthbert et al. [130], a 12 week (1 session weekly), low-volume, hamstring eccentric training programme (2 sets; 10 reps total per week) was found to be as effective as a high-volume alternative (8 sets; 40 reps total per week) in elite under-19 youth soccer players [128]. An increase in eccentric knee flexor strength and biceps femoris long head and semimembranosus FL was observed in both groups, irrespective of training volume. In contrast, Siddle et al. [46] did not find any improvements in eccentric hamstring strength or muscle architecture (muscle thickness), FL and pennation angle of the biceps femoris long head, semimembranosus and semitendinosus following an 8 week NHE intervention in a similar sample of elite youth soccer players (age 16.65 ± 0.61 years). This included 2 sessions per week for the first 2 weeks (8 sets; 48 reps total per week) before decreasing to 1 session per week for the final 6 weeks (2 sets; 8 reps total per week). The differences in findings may be explained by several methodological differences. First, whilst the NHE has demonstrated a preferential recruitment of the biceps femoris and semitendinosus [54, 131], the inclusion of a hip extension exercise (i.e. stiff-leg deadlift) [128] may have led to a more complete adaptation in muscle architecture owing to the additional recruitment of the semimembranosus [132]. Second, Siddle et al. [46] incorporated an 8 week programme that was less than the training duration, volume and intensity of Lacome et al. [128] (12 weeks) and was perhaps not sufficient to elicit adaptations of a magnitude to cause architectural changes in elite youth soccer players with greater training experience (i.e. 12 months of previous experience performing the NHE). Lastly, given that the angle of peak torque was maintained across the intervention and an approximate 3 s eccentric tempo was implemented [46], it is possible that insufficient emphasis was placed on increasing the breakpoint angle (the angle at which the individual can no longer resist the increasing gravitational moment and falls to the floor). Current evidence suggests that the NHE should be performed as slowly as possible, rather than constrained to a movement tempo, as this will encourage participants to actively hold at the upper ranges of motion before rapidly descending to the floor [133, 134]. To overcome this, future research should incorporate the breakpoint angle as a means of monitoring and progressing the NHE in youth athletes. In practice, this can be achieved via the use of validated mobile phone applications [135, 136].

Following 6 weeks of progressive NHE training (1 session per week of 2 sets of 5 repetitions increasing to 2 sessions per week of 3 sets of 6 repetitions), Drury et al. [119] noted improvements in eccentric hamstring strength in pre- and mid- to post-PHV participants. Freeman et al. [127] implemented identical sets and reps but instead included 2 sessions per week over a shorter duration (i.e. 4 weeks) as compared to Drury et al. [119]. Improvements in eccentric hamstring strength were not as evident, though the participants in this study were older (age 16.21 ± 1.34 years) [127]. During the pre-PHV years, changes in absolute stature, limb length and body mass are significantly less than at the time of PHV [12, 40, 137]; therefore, adaptations to resistance training are suggested to be as a result of improved neuromuscular activation [32, 35]. An increase in muscle strength in the absence of gains in body mass may subsequently have a greater impact on an exercise such as the NHE whereby a lesser body mass and limb length are advantageous. To further reinforce this point, Freeman et al. [127] reported absolute eccentric knee flexor strength, whilst Drury et al. [119] noted relative scores. This likely accentuated the benefits experienced by the pre-PHV participants due to their lesser body mass in addition to the accelerated adaptations previously identified in muscle architecture at this stage of maturity [108, 109]. In contrast, an increase in body mass and limb length following PHV would disguise an increase in eccentric knee flexor strength when using a relative score; instead favouring an absolute score. Through using the NordBord device, as both studies did [119, 127], it is possible to report torque about the knee joint. This would facilitate a more precise comparison between children and adolescents at different stages of growth and maturation as both force and shank length are included in its calculation. At the very least, future investigations should take maturity status and body mass into consideration to calculate a relative NHE score; however, an additional measure of knee joint torque divided by body mass would make comparisons between players of different sizes more relatable.

#### Sprint, COD and Jump Performance

Understanding the effects of a training intervention on physical performance measures such as sprinting, COD and jumping is important as these are regularly included in fitness testing and talent identification processes [138]. A total of 5 studies evaluated the effects of the NHE on measures of physical performance in youth athletes between the ages of 11 and 16 years [46, 57–59, 127] (Table 3). The results of these studies suggest that performing the NHE 1 to 3 times per week can lead to improvements in CMJ, sprint, repeat sprint ability and COD performance over the course of 6 [57] and 8 weeks [59]. However, a 4 and 6 week NHE intervention (1 to 2 sessions per week) demonstrated no improvements in sprint performance [127]. This is not surprising given the large differences in the timing, velocity, hip-knee kinematics, and overall intra- and inter-muscular coordination between an isolated hamstring exercise such as the NHE and sprint tasks [139–145], especially when included as the only intervention exercise [127]. In fact, it is recommended that the NHE is combined with regular high-speed running to elicit significant adaptations in sprint performance [146]. Additionally, the breakpoint angle was not quantified in any of these investigations; therefore, it is possible that participants simply break and lose the active muscle tension at a very early stage of the movement, which would likely nullify any potential adaptations [130]. Details of the participants additional exercise activities were also lacking in Freeman et al.’s [127] investigation, making it impossible to appreciate the impact training experience may have had upon the findings reported.

The study by Chaabene et al. [59] implemented the NHE in young female handball participants and observed improvements in a range of sprint distances (5–20 m), COD performance and CMJ height. Similarly, Hammami et al. noted an increase in sprint and COD performance in pre- and post-PHV male handball players [57] and pre-PHV weightlifters [58]. The hamstring muscles appear to be important to provide effective horizontal ground reaction force production during sprint acceleration performance [147]. More specifically, high eccentric hamstring torque capability prior to ground contact when the lower limb is in a near maximally extended position has been noted in those who produce the greatest amount of horizontal force [147], which is recognised as a key determinant of acceleration performance in youth athletes [147–151]. Furthermore, an increase in eccentric knee flexor strength may serve to help decelerate knee extension motion at the end of the swing phase during maximum speed running and realise a more powerful ground contact period [152, 153]. From an injury mitigation standpoint, the inclusion of the NHE appears to enhance hamstring muscle capacity to produce and withstand high loads at longer muscle lengths [151, 154].

Of particular importance, Chaabene et al. [59] and Hammami et al. [57, 58] included normal in-season handball training alongside the eccentric hamstring strength training intervention, which although not explicitly reported, likely included an element of high-speed running. As mentioned previously, the cumulative effect of NHE training and high-speed running is suggested to be the most beneficial towards developing sprint performance [143, 146, 154]. In addition, Hammami et al. [58] included a range of hamstring exercises as well as the NHE (single-leg Romanian deadlift [RDL], hip thrust, glute-ham raises and good morning), which may have also influenced the results. Sprinting is a multiarticular activity, whereby multiple joints and muscles contribute considerably (i.e. hip flexors and extensors) to movement execution [143]. Given that the hamstring muscles are also biarticular and the NHE is a knee dominant exercise, additional hip-dominant exercises are an essential component of an S&C programme to ensure that youth athletes experience holistic hamstring development [131, 146]. As mentioned earlier in this section, the inclusion of hip extension exercises will also lead to greater activation of the semimembranosus, while the NHE preferentially targets the biceps femoris long head and semitendinosus [131, 132, 155].

None of the investigations in this review reported negative effects on COD performance following a NHE intervention [46, 57–59, 127]. The hamstrings are considered to have an important role during the weight-acceptance phase (i.e. braking and decelerating prior to changing direction) of COD tasks in preventing anterior tibial translation and reducing anterior tibial shear and anterior cruciate ligament strain [156–159]. In further support of the current findings, eccentric hamstring strength has previously been shown to discriminate between faster and slower COD times, albeit in adult populations [91, 160, 161]. This is suggested to be as a result of the hamstrings’ role in mediating the braking forces during sudden COD tasks and the facilitation of greater hip extensor torque necessary for maintenance of trunk position and dynamic control of knee flexion, which subsequently contribute to the greater storage and utilisation of elastic energy [98, 99, 162]. Likewise, investigations have concluded that greater eccentric strength of the quadriceps or hamstrings is beneficially associated with various measures of horizontal deceleration ability (i.e. negative change in velocity, deceleration gradient, time to stop or distance to stop) [91, 162, 163]. Consequently, developing eccentric hamstring strength through the inclusion of the NHE can have a positive effect upon COD performance. Nevertheless, these studies were in adult populations and therefore, future research is needed to better understand the importance of eccentric hamstring strength in youth athletes and its contribution to physical performance tasks. It is also important to acknowledge that all the investigations in this review continued to implement their normal sport-specific training. As such, improvements in COD performance could potentially be due to a combination of the NHE intervention and a participant’s normal sport specific training, which almost certainly included high-speed running and COD activities.

Regarding CMJ performance, the knee flexors cause a forward-downward acceleration of the centre of mass, which subsequently enables the knee extensors to build up force prior to, rather than during, the concentric phase [164]. This is believed to increase muscle work and subsequent jump height and may explain the improvements noted in CMJ height following a NHE intervention [57, 59, 103, 104, 164]. The NHE may also evoke favourable adaptations in lesser trained athletes, with an increase in hamstring peak torque towards a more extended knee position suggested to offer greater knee joint stability and subsequent transfer of force during the final take-off phase (i.e. immediately prior to leaving the ground) [165]. This may have implications for optimising take-off velocity [166]. Chaabene et al. [59] reported a moderate improvement in CMJ height following an 8 week progressive NHE intervention in female youth handball participants (1 session per week of 2 sets of 5 repetitions increasing to 3 sessions per week of 3 sets of 8 repetitions). Although speculative, it was suggested that the increase in performance was related to inter-muscular coordination and greater levels of muscle activation following the NHE. However, kinetic and kinematic analyses were not undertaken, which limits the ability to accurately interpret the findings. Hammami et al. [58] investigated the effects of an eccentric hamstring strength training programme (i.e. glute-ham raise, NHE, single-leg RDL, and barbell or dumbbell good morning) on different horizontal jumping tasks in male youth weightlifting participants. Significant improvements in standing long jump and a 3 hop test were realised (Table 3), suggesting that 6 weeks of twice-weekly eccentric hamstring training is sufficient to prompt changes in horizontal jump performance. Nonetheless, given that only 2 studies were found to include measures of jump performance, further research is warranted to investigate the chronic effects of NHE on jumping tasks. For adaptations following an eccentrically dominated exercise to be appropriately interpreted using jumping tasks, it is also important that future investigations include force platform-derived eccentric phase variables to specifically identify what might have changed (i.e. braking and propulsion phase time, force, velocity, rate of force development, impulse and countermovement displacement).

Collectively, these findings have important implications for S&C coaches as they help to guide the design of NHE interventions in youth athletes. Improvements in eccentric hamstring strength seem to be mediated by alterations in muscle architecture. Specifically, and in support of similar research in adults [130, 132, 155], changes in muscle FL appear to be the main driver behind eccentric hamstring strength adaptations. This may be achieved via an increase in breakpoint angle, which causes a concurrent shift in the angle of peak torque [54, 130]. Importantly, the studies in this review demonstrated the NHE to be effective in improving jumping, sprint and COD performance in youth athletes, although this may be governed by key methodological considerations (see Sect. 3.5.2). The effects of the NHE on sprinting performance are heightened when additional high-speed running and hip extension exercises are added to youth S&C programmes, which may provide a rationale for the investigation of alternative ERT methods in addition to the NHE and those that emphasise a greater eccentric velocity [95]. Regarding youth sport, the impact of injury must be considered from the perspective of effective exercise selection. Recent findings have highlighted the prevalence of HSI in youth soccer [167] and youth athletics [168], with sprinting reported as the predominant mechanism of injury. In agreement with similar studies [169, 170], the occurrence of greater time-loss injuries increased in the older age groups (i.e. under 14 and 15 years of age groups), with greater severity of injury in the under 16 years of age group [167]. Together, these results suggest that S&C programmes, inclusive of ERT methods such as the NHE, should begin at an earlier age (i.e. approximately 10–12 years or the age at which a child can follow instructions in the context of organised training), to allow time to develop the requisite training age and technical competency. A focus on holistic hamstring development may subsequently help to mitigate injury occurrence as children experience growth and maturation.

### Chronic Effects of FIT on Physical Performance

The current review found FIT interventions to be the most frequently utilised ERT method in youth athletes (Table 4). These investigations incorporated several different measures to quantify physical performance before and after a FIT intervention, including sprint speed [171–175], COD performance [171, 174–176], NHE breakpoint angle [43], isometric strength [175], and numerous jumping tasks and metrics [43, 171–175, 177, 178]. The participants in these studies had a mean ± standard deviation age of 15.8 ± 2.0 years (range 11.8–18 years).

#### Sprint and COD Performance

Of the 5 studies that measured sprint speed, 3 found improvements following FIT over distances that ranged from 5 to 60 m [171, 172, 175]. Fiorilli et al. [171] undertook a 6 week FIT intervention (2 sessions per week) and compared this to a plyometric training group in male soccer participants. The FIT exercises included a diagonal sprint with the cable attached to the waist (4 sets of 7 repetitions), and a simulated soccer shot with the cable attached to the ankle. Participants were encouraged to perform the concentric phase maximally and resist the eccentric phase whilst back pedalling. Although the most beneficial effects were noted in COD performance, significant improvements were also demonstrated in 60 m sprint time in comparison with the plyometric training group. Similarly, de Hoyo et al. [172] and Stojanović et al. [175] implemented a range of FIT exercises, including a prone leg curl and half-squat [172, 175], RDL, one-arm row, rotational Paloff press and biceps curl plus upright row [175] 1 to 2 times per week for 10 and 8 weeks, respectively. Both investigations demonstrated improvements in sprint speed over 5–20 m, suggesting that a range of FIT exercises can be used to enhance sprint performance in youth athletes (refer to Table 4).

It appears that when FIT is implemented in younger participants (i.e. < 12 years of age), improvements in sprint performance are not so clear. Westblad et al. [173] compared an autoregulated FIT squat and a traditional free-weight barbell squat in pre-PHV athletes over the course of 6 weeks. Both groups improved CMJ and SJ performance without any significant differences in sprint times from pre- to post-intervention. Similarly, Raya-González et al. [174] investigated the effects of a FIT lateral squat over 10 weeks in an under-16  age group of soccer players and found no improvements in sprint time over 10, 20 and 30 m. Nonetheless, the training intervention led to an increase in CMJ and COD performance. One important difference between these findings and those above [171, 172, 175] is that in the study by Westblad et al. [173], progressions in intensity were achieved via autoregulation. Although the rationale behind autoregulation is to prevent excessive fatigue and therefore, incur greater adaptations [179], caution must be taken to ensure that appropriate supervision is in place, especially in children as young as those in this investigation (mean age 11.8 years) [173]. This is to ensure that the correct technique is adopted, and any potential risk of injury is mitigated [3, 36] Further research is also necessary to provide specific guidelines to help S&C coaches implement autoregulated resistance training in practice.

Regarding FIT intensity, previous research in adult populations has demonstrated lower inertial loads (i.e. 0.025–0.050 kg·m^2^) to be appropriate when the objective is to promote a higher movement velocity and power output [48, 180, 181], whereas higher inertial loads (i.e. > 0.050 kg·m^2^) may be more suitable to develop strength-related characteristics [182, 183]. In the study by Westblad et al. [173], inertial loads of between 0.025 and 0.050 kg·m^2^ were prescribed, which was likely too great for this population, especially when considering that other investigations have recently demonstrated the inability of young athletes to translate the energy absorbed during the eccentric phase of jump tasks into concentric performance [111, 184]. Another key consideration when interpreting the differences is the direction of force applied during the FIT exercise. That is, Fiorilli et al. [171] utilised FIT exercises that emphasised horizontal force application to simulate sprint acceleration. Likewise, de Hoyo et al. [172] and Stojanović et al. [175] included additional exercises that possibly complemented the FIT half-squat (i.e. hamstring exercises), despite it being a vertically dominant exercise. Previous investigations support this finding and reinforce the effect that movement-specific strength training can have on measures of physical performance [185], whilst emphasising the theory of dynamic correspondence (i.e. the ability of an exercise or programme to directly impact the athlete’s sporting performance) [97, 140, 186].

Although COD performance is improved following FIT exercises that emphasise lateral movement [171, 174], the current findings suggest that enhancements can also be made following a training programme including FIT exercises that focus on vertical force application [175, 176]. This contrasts with previous recommendations that have suggested movement-specific strength training to be key to experiencing beneficial effects on physical performance tasks [97, 140]. The enhancements could be related to the similarities between FIT and COD tasks [187]. That is, FIT appears particularly effective for simulating the repeated rapid braking and propulsive actions experienced when performing COD tasks, particularly when the overload is resisted during the final one- to two-thirds of the eccentric phase [45, 182, 188]. In support of this, Berg and Tesch [189] reported the most effective FIT technique to maximise eccentric overload is to gently resist the force during the first third of the eccentric phase before maximally decelerating the rotating flywheel. Furthermore, an 8 week FIT programme, including half-squats and RDLs, demonstrated large improvements in COD performance [175]. Likewise, de Hoyo et al. [172, 176] found substantially greater braking and propulsive force and impulse and reduced contact times following 10 weeks of FIT half-squats and prone leg curls. These outcomes support investigations that have suggested increases in braking force and impulse to subsequently enhance propulsive performance via an increase in the storage and utilisation of elastic energy [190]. Therefore, COD performance can be improved via both horizontally and vertically dominant FIT exercise in youth populations. However, it is worth reiterating that appropriate familiarisation (i.e. a minimum of 3 sessions) and technical proficiency are the key prerequisites for effective FIT adaptations and outcomes [67, 92, 174, 191]. Indeed, those with more FIT experience exhibit a significantly greater eccentric and concentric peak force output [192]; therefore, a detailed report of participants training status and experience is essential in future FIT research.

#### Jump Performance

Jumping performance is frequently used to provide an indication of neuromuscular function and physical capacity in both adult and youth athletic populations [19, 111, 193, 194]. The findings of this review suggest that jump height can be enhanced following the completion of a FIT intervention [43, 69, 171, 173–175, 177, 178] (Table 4), which agrees with previous evidence [81, 187]. The largest improvements were found following FIT exercises that emphasise vertical force production (i.e. FIT half-squat) [172, 175, 177]. Similarly, horizontal jump, lateral jump and diagonal single-leg rebound jump distances were improved to the greatest extent following training interventions that include FIT exercises with more of an emphasis placed on horizontal or lateral force production (i.e. reverse lunge, side-step and eccentric resisted sprints). This further reinforces the suggestion that adaptations observed following FIT are movement specific [185].

The efficacy of FIT to improve jump performance is best explained by the mechanical nature of this device. That is, the participant accelerates the flywheel (resistance due to the flywheel moment of inertia) with maximal effort during the concentric phase of the movement, resulting in flywheel kinetic energy and inertial torque that imparts high linear resistance during the subsequent eccentric phase [195, 196]. This may provide an optimal stimulus for adaptations related to jumping performance, such as improvements in the SSC and muscle–tendon unit stiffness. Arede et al. [177] implemented a 6 week FIT programme (2 sessions per week) consisting of backward lunges, shuffling steps and side steps performed under normal and variable conditions (i.e. participants were instructed to move in different directions randomly at the start of each concentric phase) in youth female team sport athletes. Significant improvements were noted in vertical and horizontal jumping in both conditions, although single-leg CMJ performance was enhanced to a greater extent following the variable FIT condition. The nature of this condition placed an increased need for lower-limb stabilisation around the primary muscles, which may have subsequently led to greater input from the associated musculature. Significant improvements in DJ, repeated hop and SJ height following 6 weeks of diagonal sprints using a FIT device have also been reported [171]. Importantly, these results were not supported by improvements in contact time or RSI scores, suggesting that the FIT intervention may have led to a different jump strategy whereby participants spent longer applying force during both the braking and propulsive phases. Following 10 weeks of a FIT lateral squat in under-16 soccer players, Raya-González et al. [174] observed significant improvements in single-leg CMJ height, but only in the non-dominant limb. This is likely best explained by the identical load that was used between limbs providing an adequate stimulus for the non-dominant side whilst neglecting the dominant side.

Whilst some of the investigations found improvements in CMJ performance following FIT [43, 172, 175], others did not [173, 178]. A few methodological differences may explain these findings. First, when participants are instructed to resist during the final one- to two-thirds of the eccentric phase, improvements in CMJ performance appear to be greater [43, 172, 175]. Second, Murton et al. [178] encouraged self-selected rest periods that need to be supervised by suitably qualified S&C practitioners to ensure that an appropriate rest period is taken in line with the desired adaptation. Although younger children may in fact recover quicker than adolescents and adults, previous research has highlighted the inability of less mature youth athletes to regulate their performance when using self-selected rest periods [197, 198].

Westblad et al. [173] also included pre-PHV participants who were unfamiliar with resistance training and therefore did not have the necessary experience or technical proficiency to realise improvements following FIT [199]. This is further confounded when considering the inertial loads implemented (0.025–0.05 kg·m^2^), as mentioned previously (see Sect. 3.6.1). Large multi-joint exercises such as a squat or an RDL may also allow participants to halt higher inertial loads and therefore achieve a more significant eccentric overload compared with single-joint movements such as a prone leg curl [200]. Resisting over the entire eccentric phase appeared to favour adaptations in SJ performance [173, 178], suggesting that this approach may be more optimal for exercises requiring a longer isometric phase in a greater range of knee flexion. In contrast, resisting for the last one to two thirds of the eccentric phase is suggested to provide a greater eccentric overload because the entire propulsive effort is resisted over a much shorter time period [180]. This strategy could have also resulted in a greater active state and number of cross-bridge attachments [103–105]. Collectively, it appears that the mechanical nature of the eccentric phase during FIT leads to a more effective braking strategy, whereby participants develop a better ability to utilise kinetic energy as elastic potential energy that is used to enhance the propulsive action. Therefore, S&C coaches should carefully consider the desired adaptation before designing and implementing FIT in youth athletes. Furthermore, the FIT studies in this review that have included a jumping assessment as a pre- and post-intervention measure of performance have reported jump height, RSI and ground contact time only [43, 69, 171, 173, 175, 177]. Given that one of the main rationales for the inclusion of FIT is to target the improvement of eccentric phase characteristics (i.e. force), it is important that future research reports force platform-derived eccentric phase variables in their analysis for better understanding of the changes observed.

#### Strength Performance

Only one study in the current review directly investigated the effects of a FIT training programme on muscular strength [175]. Stojanović et al. [175] implemented an 8 week FIT intervention in comparison with a free-weight training group, both consisting of 1 to 2 sessions per week and a control group who continued their normal basketball training routine only. A half-squat (i.e. 100 degrees of knee flexion) isometric strength assessment was conducted pre- and post-intervention. Both intervention groups experienced a substantial improvement in isometric strength, whilst the control group only realised a small effect (refer to Table 4). Given that the effects on strength were greater in the FIT condition compared with the free-weight intervention, the nature of the FIT half-squat may explain these differences. More specifically, participants were required to execute the concentric phase with maximal effort before applying maximal force after the first third of the eccentric phase, in order to stop the flywheel at 90 degrees of knee flexion. Given that the isometric strength assessment was carried out at 100 degrees of knee flexion and the FIT device would have imparted an eccentric overload at approximately this range of motion, the benefits gained from the FIT device were specific to that of the subsequent strength assessment. Information pertaining to the free-weight condition was lacking; however, it is possible that participants would have reached a similar squat depth, although the eccentric effort would have been distributed over the entire phase, rather than concentrated in the final two thirds. Although not reported, the strategy employed in the FIT condition would have demanded a greater braking impulse ( the product of force and time) to stop the movement [201, 202], resulting in a greater force output at the bottom of the squat that was carried into the propulsive action.

Di Cagno et al. [43] assessed the NHE breakpoint angle pre- and post-intervention following 6 weeks of progression FIT exercise in male fencing participants to provide an insight into eccentric hamstring strength. The intervention included variations of a fencing-specific lunge with the FIT device placed behind or in front of the participant. There were no significant improvements found in the NHE breakpoint angle, which support previous review guidelines that suggest lunge exercises can be used to improve gluteus muscle but not hamstring strength [203, 204]. Furthermore, the NHE is an eccentric dominant exercise that requires specific training to elicit meaningful adaptations. Thus, exercise selection should be carefully considered before implementing if the objective is to enhance the NHE breakpoint angle, with FIT lunge variations being more suited to improving fencing-specific movements [43]. To improve the current literature, future work is necessary to understand the impact of different inertial loads on muscular strength adaptations and the most effective dose–response relationship in youth athletes across maturity groups.

### Chronic Effects of Alternative ERT Methods on Physical Performance

Other than NHE and FIT training programmes, only 2 studies were included in the current review that implemented an alternative ERT strategy [205, 206] (Table 5). Both studies included an emphasis on tempo training (i.e. manipulation of the speed or time of a given exercise) with a range of strength and COD assessments used to quantify changes in physical performance. The mean age of participants in these studies was 16.4 ± 1.9 years (range 15.0–17.7 years).

Bourgeois et al. [205] implemented a variety of free-weight exercises, including a parallel back squat and hexagonal-bar squat, 3 times a week for a total of 6 weeks. Exercises were either performed with a 3 s eccentric duration (eccentric group) or with no tempo constraints (control group). The control group achieved the greatest improvements in a 180 and 45 degree COD approach and exit times whilst also realising a greater acute enhancement in unilateral isometric strength. In contrast, the eccentric group displayed a better ability to retain and further improve their unilateral isometric strength, which agrees with previous research [5, 38, 39, 69, 176, 207]. The greater improvements found in 180 degree COD performance in the eccentric training group suggest that a COD of this magnitude necessitates a greater emphasis upon weight acceptance, braking and propulsive forces [89, 90, 190]. In support of this, 45 degree COD performance was negatively affected in the eccentric group, suggesting that tempo training lacks specificity to a COD angle whereby limited braking is required as instead the maintenance of velocity is crucial [98, 208]. Hence, a faster training mode (i.e. jump-based AEL, accelerated eccentrics and accelerated eccentric loading, see Handford et al. [95] for a review of these methods) may be more beneficial for improving 45 degree COD tasks [95, 209]. These findings have important implications for the development of training programmes for youth athletes and should be verified in future investigations.

Dafkou et al. [206] recently investigated the effects of an 8 week training programme with 2 sessions per week, consisting of a single-leg sliding hamstring curl and a variety of single-leg balance and core exercises. Initially, 2 sets of 6 repetitions were performed on each leg increasing to 4 sets of 10 repetitions by week 6, all with a 6 to 8 s eccentric tempo. Although improvements in eccentric hamstring strength were demonstrated, these were small and insignificant, which is contrary to previous investigations [210–212]. This is best explained by the differences in training volume and participants between studies. Specifically, only 1 eccentric hamstring exercise was included in this study and the participants were semi-professional soccer players with a wealth of sport-specific training experience. Given that the sliding hamstring curl is a bodyweight exercise, it likely lacked the ability to provide sufficient progressive overload throughout the training programme to elicit greater developments in muscular strength [21, 37, 213]. The participants pre-intervention eccentric strength values were also approximately 1.5–3 times greater than those reported by previous studies [210, 214, 215] that have found more beneficial effects. Lastly, both groups continued with their normal soccer training that included aerobic, plyometric, sprint and endurance training, which would have interfered with the training adaptations noted [216–218]. Therefore, future studies should look to build upon these findings with the inclusion of additional strength exercises to create a comprehensive S&C programme.

## Conclusions

This review has highlighted ERT methods such as the NHE, FIT and tempo training to be effective in enhancing measures of physical performance (i.e. jump, sprint, COD and strength tasks) in youth athletes. However, there are some considerations to be made before incorporating these methods into S&C practice. The effects of FIT on acute PAPE effects are currently limited to 2 studies, although these have demonstrated promising results. These results appear to be somewhat limited by the training experience of participants, with the inclusion of adequate familiarisation being essential. The kinetic analysis of dumbbell AEL DJs was also positive, although the combination of a new training method (AEL) and a technically challenging exercise (DJ) appear to have contributed to the extensions in ground contact time and reductions in spring-like behaviour observed. Nevertheless, meaningful improvements in jump height were noted, which suggests that future research should investigate the effects of dumbbell AEL on a less technically challenging exercise (i.e. CMJ). The chronic NHE findings have shown that an emphasis should be placed on progressing intensity via an increase in breakpoint angle whilst maintaining, rather than increasing, training volume to prioritise movement quality and eccentric hamstring strength development. Improvements in sprint speed and COD performance appear to be enhanced when the NHE is combined with high-speed running. Following chronic exposure to FIT, this review found improvements in several measures of physical performance (i.e. jumping, sprinting, COD and fencing-specific lunge movements); however, it is recommended that participants are instructed to resist the inertia developed during the concentric effort in the last one- to two-thirds of the eccentric phase to ensure the greatest chance of an eccentric overload. Eccentric tempo training is another viable method that has been found to elicit improvements in COD performance and strength characteristics. Given that the NHE and FIT were the most popular ERT methods found in this review, future research should explore the efficacy of alternative methods in youth athletes (i.e. jump-based AEL methods).

## Declarations

### Availability of data and material

The data within this study are secondary data and available through the relevant articles referenced throughout and in the References list.
